# Supplementary material for: Improving Peer Online Forums (iPOF): protocol for a realist evaluation of peer online mental health forums to inform practice and policy
Source: BMJ Open. 2023 Jul 30;13(7):e075142. doi: 10.1136/bmjopen-2023-075142 (PMC10387651; doi:10.1136/bmjopen-2023-075142)
Supplement: Author's manuscript [file bmjopen-2023-075142.draft_revisions.pdf]

BMJ Open

BMJ Open is committed to open peer review. As part of this commitment we make the peer review history of every article we publish publicly available.

When an article is published we post the peer reviewers' comments and the authors' responses online. We also post the versions of the paper that were used during peer review. These are the versions that the peer review comments apply to.

The versions of the paper that follow are the versions that were submitted during the peer review process. They are not the versions of record or the final published versions. They should not be cited or distributed as the published version of this manuscript.

BMJ Open is an open access journal and the full, final, typeset and author-corrected version of record of the manuscript is available on our site with no access controls, subscription charges or pay-per-view fees (http://bmjopen.bmj.com).

If you have any questions on BMJ Open's open peer review process please email info.bmjopen@bmj.com

Improving Peer Online Forums (iPOF): Protocol for a Realist Evaluation of Peer Online Mental Health Forums to Inform Practice and Policy

Journal:	*BMJ Open*
Manuscript ID	bmjopen-2023-075142
Article Type:	Protocol
Date Submitted by the Author:	27-Apr-2023

Complete List of Authors:	Lobban, Fiona; University of Lancaster, Division of Health Research<br>Coole, Matthew; Lancaster University<br>Donaldson, Emma; Berkshire Healthcare NHS Foundation Trust<br>Glossop, Zoe; Lancaster University, Spectrum Centre for Mental Health Research<br>Haines, Jade; Berkshire Healthcare NHS Foundation Trust<br>Johnston, Rose; Lancaster University, Spectrum Centre for Mental Health Research<br>Jones, Steven; Lancaster University, Spectrum Centre for Mental Health Research, Division of Health Research, Faculty of Health and Medicine<br>Lodge, Christopher; Lancaster University, Spectrum Centre for Mental Health Research<br>Machin, Karen; Independent Survivor Researcher<br>Marshall, Paul; Lancaster University, Spectrum Centre for Mental Health Research, Division of Health Research<br>Meacock, Rachel; University of Manchester<br>Penhaligon, Kate; Berkshire Healthcare NHS Foundation Trust<br>Rakić, Tamara; Lancaster University<br>Rawsthorne, Mat; Service User Researcher<br>Rayson, Paul; Lancaster University<br>Robinson, Heather; Lancaster University<br>Rycroft-Malone, Jo; Lancaster University<br>Semino, Elena; Lancaster University, Linguistics and English Language<br>Shryane, Nick; The University of Manchester School of Social Sciences, Social Statistics<br>Wise, Sara; Berkshire Healthcare NHS Foundation Trust

Keywords:	MENTAL HEALTH, Patient Participation, QUALITATIVE RESEARCH, HEALTH ECONOMICS

SCHOLARONE™
Manuscripts

Improving Peer Online Forums (iPOF): Protocol for a Realist Evaluation of Peer Online Mental Health Forums to Inform Practice and Policy

Author Information

Fiona Lobban, Lancaster University, f.lobban@lancaster.ac.uk, ORCID: 0000-0001-6594-4350

Matthew Coole, Lancaster University, m.coole@lancaster.ac.uk, ORCID: 0000-0002-9279-931X

Emma Donaldson, Berkshire Healthcare NHS Foundation Trust, Emma.Donaldson@berkshire.nhs.uk, ORCID: 0000-0003-0304-8360

Zoe Glossop, Lancaster University, z.glossop@lancaster.ac.uk, ORCID: 0000-0002-6151-4860

Jade Haines, Berkshire Healthcare NHS Foundation Trust, jade.haines@berkshire.nhs.uk

Rose Johnston, Lancaster University, r.johnston2@lancaster.ac.uk, ORCID: 0000-0002-6863-2624

Steven Jones, Lancaster University, s.jones7@lancaster.ac.uk, ORCID: 0000-0002-8801-5113

Christopher Lodge, Lancaster University, c.lodge@lancaster.ac.uk, ORCID: 0000-0003-1115-5553.

Karen Machin, Independent survivor researcher, karen@role.org.uk, ORCID 0000-0002-0374-4238

Paul Marshall, Lancaster University, p.marshall4@lancaster.ac.uk, ORCID: 0000-0002-3913-3359

Rachel Meacock, The University of Manchester, rachel.meacock@manchester.ac.uk, ORCID: 0000-0001-8933-5058

Kate Penhaligon, Berkshire Healthcare NHS Foundation Trust, kate.penhaligon@berkshire.nhs.uk

Tamara Rakić, Lancaster University, t.rakic@lancaster.ac.uk, ORCID: 0000-0002-8591-923X

Mat Rawsthorne, Service User Researcher, mat.rawsthorne@proton.me, ORCID 0000-0002-7481-693X

Paul Rayson, UCREL, School of Computing and Communications, Lancaster University, p.rayson@lancaster.ac.uk, ORCID: 0000-0002-1257-2191

Heather Robinson, Lancaster University, h.robinson@lancaster.ac.uk, ORCID: 0000-0002-3566-2253

Jo Rycroft-Malone, Faculty of Health & Medicine, Lancaster University, j.rycroft-malone1@lancaster.ac.uk, ORCID: 0000-0003-3858-5625

Elena Semino, Department of Linguistics and English Language, and ESRC Centre for Corpus Approaches to social Science, Lancaster University, e.semino@lancaster.ac.uk, ORCID: 0000-0002-3421-2963

Nick Shryane, Department of Social Statistics, University of Manchester, N.Shryane@manchester.ac.uk, ORCID 0000-0001-7963-4601

For peer review only

Sara Wise, Berkshire Healthcare NHS Foundation Trust, sara.wise@berkshire.nhs.uk

Funding Statement

This study is funded by the NIHR Health and Social Care Delivery Research (HS&DR) (NIHR134035). The views expressed are those of the author(s) and not necessarily those of the NIHR or the Department of Health and Social Care. The study is hosted by Berkshire Healthcare NHS Foundation Trust. The Sponsor is Lancaster University. Additional support comes from the NIHR Clinical Research Network, NIHR North-West Coast Applied Research Collaboration (ARC), and Oxford Allied Health Sciences Network (AHSN).

Competing Interests statement

The authors declare no competing interests.

Word Count

4775

For peer review only

Abstract

Introduction. Peer online mental health forums are commonly used and offer accessible support. Positive and negative impacts have been reported by forum members and moderators, but it is unclear why these impacts occur, for whom, and in which forums. This multiple method realist study explores underlying mechanisms to understand how forums work for different people. The findings will inform codesign of best practice guidance and policy tools to enhance the uptake and effectiveness of peer online mental health forums.

Methods and Analysis. In workstream 1 we will conduct a realist synthesis, based on existing literature and interviews with approximately 20 stakeholders, to generate initial programme theories about the impacts of forums on members and moderators and mechanisms driving these. Initial theories that are relevant for forum design and implementation will be prioritised for testing in workstream 2.

Workstream 2 is a multiple case study design with mixed methods with several online mental health forums differing in contextual features. Quantitative surveys of forum members, qualitative interviews, and Corpus-based Discourse Analysis and Natural Language Processing of forum posts will be used to test and refine programme theories. Final programme theories will be developed through novel triangulation of the data.

Workstream 3 will run alongside workstream 1 and 2. Key stakeholders from participating forums, including members and moderators, will be recruited to a Codesign group. They will inform the study design and materials, refine and prioritise theories, and codesign best policy and practice guidance.

Ethics and dissemination. Ethical approval was granted by Solihull Research Ethics Committee (**IRAS 314029**). Findings will be reported in accordance with RAMESES (1) guidelines, published as open access and shared widely, along with codesigned tools.

Registration details. The study is prospectively registered at ISRCTN 62469166. The protocol for the realist synthesis in workstream one is prospectively registered at PROSPERO CRD42022352528.

Strengths and limitations

- A detailed methodology is presented to develop and test a programme theory about how and why online forums can support people with mental health challenges and their carers. This methodology may inform the design of future studies to evaluate complex online interventions which are already in widespread use.
- A novel triangulation of survey, interview and corpus linguistic methods is described, that integrates the analysis of primary and secondary data sources, requiring collaborative working between interdisciplinary researchers with varying levels of experience in realist approaches.
- The process by which realist programme theory can be used to inform the codesign of best policy and practice guidance with people with lived expertise is outlined. This demonstrates the role of Patient and Public Involvement (PPI) in bridging the gap between theory development and intervention design.
- Ethical issues of using online forum posts in research are identified, and a framework for addressing these will be produced.

For peer review only - http://bmjopen.bmj.com/site/about/guidelines.xhtml

Introduction

Mental health problems affect approximately 1 in 6 people in the UK at any one time (>11million people) (2). People seek support in a variety of ways, increasingly through peer support and by going online (3, 4). They search for information and join online mental health forums: dedicated support platforms aimed at helping individuals to share, discuss and solicit information and support (5). Many are moderated, and some have access to additional online tools and resources. Although widely used, the impacts of using online forums, and mechanisms by which these occur, are poorly understood.

Online forums can provide round-the-clock opportunities for members to feel understood, access information, make friends, and use their experiences to help others (6) whilst being able to manage how they present themselves online (7-9). These forums can function as an important gateway to other offline support as people test out sharing their problems and are encouraged to access help (10-13). Online forums are particularly valued by people with rare or highly stigmatised difficulties, and those living in remote areas, or close knit communities wanting anonymity (14). Online forums offer robust support during pandemics when healthcare services are restricted, and for people in low- and middle-income countries where access to mobile technology is common but healthcare is not. Studies have shown positive outcomes associated with using online forums, including reduction in depression (15), suicidal thoughts (16), and social isolation (14).

However, negative impacts of using online forums have also been reported including increases in suicidal ideation (17), more negative mood (18), and worsening of body image and disordered eating patterns (19). Forums carry inherent risks of disinformation, dependency, and sharing of unhelpful strategies. Information shared can be misleading, inaccurate, harmful, or triggering(6, 20). Hearing about the experiences of others can exacerbate low mood and risk of harms (21) and generate unrealistic expectations and greater anxiety or confusion about one's own condition (22). Normative ideas about health may, leave some users feeling misunderstood or even bullied, and people in mental distress may be particularly vulnerable to the negative impacts of this kind of behaviour (23). Healthcare staff may be reluctant to moderate online forums because of concerns about possible harms and lack of protocols for online working (24), and may be reluctant to recommend specific forums without clearer guidance on criteria by which to make this decision (25).

Current research suggests a wide range of impacts from using online mental health forums, but with little understanding of why these vary across individuals (14-20). Particular features of forums such as size and heterogeneity, target population, ease of access and level of friction, how individual identities are managed, forum rules, and incentives for use and for pro-social behaviour have all been hypothesised as features that might account for the variation in impacts (26-29). Style of moderation is also likely to be important. The moderator role is highly variable across forums, ranging from identifying and removing risk-related materials, to that of a highly skilled online counsellor, directing the focus and values of the online forum (30).

Understanding how these contextual features influence the culture and function of forums is crucial. Hence, research needs to go beyond the study of behaviour in individual forums and investigate what aspects of design, moderation, and culture differ between forums, which facilitate better outcomes, and how (26, 31). Research to date has drawn on quantitative surveys, qualitative interviews, computational discourse analysis, and social network analysis, but all have limitations when used in isolation. Our research will move the field from single forum and single methods studies, to the triangulation of findings from a range of methods and online forums, sampled for contextual diversity, to test comprehensive programme theories. The programme theories will then be used to inform co-design of best practice and policy guidance with the aim of maximising positive impacts of online forums, whilst minimising risks of any negative impacts on users and moderators.

For peer review only

Aims and Objectives

Aims

To develop a programme theory to explain the underlying mechanisms by which online mental health forums impact on people's mental health and wellbeing.
To use this programme theory to develop best practice tools to improve uptake, safety and usefulness of online forums.

Objectives

Objectives are aligned to specific research questions (RQ).

RQ1: What are the impacts of using online mental health forums for people experiencing mental health difficulties? How, why, in what contexts, and for whom are these impacts generated?
Objectives
1a. Develop theories of the underlying generative mechanisms by which, and contexts within which, online mental health forums impact on mental health and wellbeing outcomes for members.
1b. Test and refine the theories in case studies of online mental health forums.

RQ2. What are the roles of forum moderators and how do they impact online mental health forums?
Objectives
2a. Develop a theory to understand the roles of online mental health forum moderators, how they vary in different contexts, and how they impact forums.
2b. Test and refine the theories in case studies of online mental health forums.

RQ3. How can evidence-based theories of online mental health forums be used to inform best practice guidance and support?
Objective
3a. Co-produce best practice tools, training and implementation plan to optimise the design and delivery of online mental health forums.

Methods and Analysis

Study design

A realist informed mixed methods evaluation, including multiple case studies (32) to co-design theory informed best practice guidance tools for online mental health forums.
Date collection will start in March 2022 and finish in December 2024

Figure 1 provides an overview of the study design.

[Figure 1. iPOF Study Design here]

Setting

Our research takes place online. Our cases are mental health forums, defined as:
online spaces to facilitate peer to peer sharing, which are aimed at young people and adults in the UK, and set up by a UK-based healthcare provider, charity, or commercial organisation with the explicit purpose of facilitating peer to peer support for people with mental health difficulties. Although people talk about mental health online in a variety of online spaces (including Facebook, Twitter, Instagram etc.), we have focussed on UK-based forums set up specifically to support mental

For peer review only

health, because we hypothesise that the impacts of forums will be strongly influenced by their broader social context, including access to other health and social care services.
We aim to recruit 6-8 forums, purposively sampled for diversity across host, target population, design (including level and nature of moderation, and whether they require registered login), and size of population. Forums will be identified using snowballing techniques and online searching. We aim for maximum variation in our sample.

Patient and public involvement (PPI) statement

Pre-funding, we hosted two online PPI events, each with twelve people with lived experience of moderating and/or regularly using online forums. Moderators reported significant challenges in their roles, impacting on their own mental health, and were very keen to receive better training and support. Forum members identified the need for healthcare staff and commissioners to be more aware of the role online forums can play in mental health support, and the need to understand the role of "super-users" who post a lot, and "observers" who read but do not post.

Our project management team includes people with lived experience of using online resources to manage mental difficulties guiding the design and delivery of the project.

We will establish a PPI/Codesign group consisting of forum members and moderators from participating forums. We will recruit widely across age, gender, ethnicity, disability, and digital engagement. The group will meet online monthly, facilitated by our PPI leads, and will contribute to the design and materials for the study, prioritise and refine programme theories, and codesign key outputs.

We also have two people with lived experience of using online mental health forums as independent members of our Study Steering Committee, meeting approximately every 6 months, to oversee the conduct of the study on behalf of the funder.

All PPI members will be invited to whole team training at the launch of the study to support a working understanding of all research methods drawn on in this study. Individual needs for training and support will be regularly reviewed and provided from across the team.
PPI will be reported in the final paper according to the GRIPP2 reporting checklist (33).

Methods

The study is organised in three parallel workstreams.

Workstream 1 (1-18 months). Theory building and hypothesis generation

Following established realist methodology (34-36), we will develop our initial candidate theories of how online mental health forums "work" (**RQ1a**) by drawing on well-established theories (37) reviewing relevant literature (including grey literature and intervention protocols), and conducting in-depth interviews with key stakeholders. The importance of forum moderation on outcomes is well established (6, 14, 30, 38), but not well understood. Therefore, to answer **RQ2a** we include a nested review question focussing specifically on understanding the role of the moderator and how this works in different contexts.

We will use the framing of If-then Context-Mechanism-Outcomes (CMO) configurations to guide all stages of the process, from theory construction to the development of data collection protocols and

For peer review only

data analysis. Outcomes will include positive and negative impacts on forum member emotional wellbeing or functioning, such as anxiety, mood, personal recovery, quality of life, and help seeking. Mechanisms will be defined as the responses of the forum members to the forum resources (including moderation) such as safety, toxicity, empathy, normalisation, bullying or reframing. Context will be explored at several levels and include any features of the forum, or backdrop against which it is being delivered, or the individual members, that may influence these mechanisms e.g. hosting, design, size, target population, or anonymity.

We will first develop an initial framework for candidate CMOs based on existing formal theories. Selection will be guided by the extent to which each theory addresses our research aims, and their compatibility with the realist approach and underlying philosophy i.e. to what extent they help to identify generative causal explanations for key outcomes.
Next, with additional support from our information specialists at Lancaster University Library, we will develop a strategy to review existing literature in relevant electronic databases such as Medline, PsychInfo, EMBASE etc., and additional grey literature (using Google, TRIP, overton.io, ProQuest, Allcatsrgrey, the International Clinical Trials Registry, and the NHS Knowledge and Library Hub) that report on the contexts in which online mental health forums are offered; the intended / proposed / measured outcomes; and explicit / implicit theories about how they "work". The search strategy will be systematic and clearly documented but will develop iteratively to include more diverse data sources that can shed light on generative causal pathways to outcomes. Data sources will include primary data, reviews, policy documents, training manuals, theoretical commentaries, author interpretations, websites and unpublished reports that give a rationale for offering online mental health forums, describe or evaluate online mental health forums, or detail the lived experiences of members/moderators/hosts/commissioners. Screening (initially by title and abstract, with 10% checked by a second reviewer) will be based on functional criteria of relevance, rigour and richness i.e. whether the document tells us anything about contexts, mechanisms, or outcomes of interest, rather than methodological quality (34). Data extraction will focus on extracting explanatory accounts and mapping where this data is coming from (the context, nature of the intervention etc.). In parallel to the literature review, we will interview up to 20 stakeholders including academics, clinicians, commissioners, policy makers, and moderators with broad expertise in online mental health forums. Sampling will be done through snowballing techniques, drawing on the networks of our broad research team, and key individuals identified in our literature searching. Data collection will be done flexibly (to maximise participation) using individual/ group interviews, online / face to face /telephone, and using topic guides designed to elicit CMO explanatory theories.

Analysis of literature and interview transcripts will be conducted using retroductive reasoning i.e. working back from the data to identify the context-dependent mechanisms underlying the impacts described in online forums. Particular attention will be paid to accounts of potential negative impacts ("dark logic models"(39)) and those that are unexpected or challenge our initial programme theories.

We will articulate a theory of how peer online forums impact forum members and moderators, starting with an array of potential outcomes, multiple possible mechanisms, and an initially broad context. We will work with our PPI/Codesign Group through prioritisation and consensus building, to ensure our final explanatory theory is comprehensible, testable, and useful for the design of policy and practice tools in WS3.

Workstream 2 (Month 6-30) Theory testing and refinement

In Workstream 2, hypotheses about 1) how online mental health forums work for whom, how and in what context (**RQ1b**), and 2) the role of forum moderators (**RQ2b**), will be tested in a longitudinal realist evaluation using novel triangulation of survey, interview, and corpus linguistic methods.

We will first develop a detailed case report for each forum describing key contextual features (based on aggregate data provided by the host) identified in Workstream 1 that influence user experiences e.g., design of forum (including access to additional online tools), ease of access and level of friction, size and heterogeneity of user population, how individual identities are managed (user identification vs anonymity), forum rules, and incentives for use and for pro-social behaviour. We will describe moderation strategies in each case including how moderators are recruited, trained and supported, moderating rules and how they are developed, temporality, transparency and opportunities for redress to moderation decisions. We will describe the linguistic properties of each forum in these case descriptions, focussing on the differences between the forums in terms of theme, topics of discussion, semantic categories, sentiment and emotion categories, and keywords.

Consistent with optimal case study design and realist approaches, we will take a mixed-method approach to explore relationships between quantitative measures of outcomes, contextual factors and some mediators, combined with an in-depth qualitative exploration of processes underpinning the deeper causal relationships between these variables. This will ensure that demi-regularities between variables across the sample are not in themselves assumed to be causal.

Surveys assessing impacts within and across cases

Surveys of forum users will quantitatively assess impacts of forum use, and evaluate potential mediators and moderators, at baseline, six weeks, and 12 weeks. The choice of these intervals is based on times to clinically important change observed in previous online peer-support studies (40, 41). Up to two iterations of the survey will allow for additional adaptation of measures should new hypotheses arise.

Participants will be recruited over a 9-month period across all forum cases, and will include members who passively view but rarely or never actively post to the forums (observers). Invitations and a link to the survey will be shared by forum hosts. On following that link, potential participants will see a participant information sheet (PIS), consent form, and only after providing their consent, the survey measures hosted on REDcap (42) to facilitate linkage between survey and forum use data participants will be invited to provide their username if they wish, and to indicate if they are willing to be contacted for an in-depth interview. To ensure user anonymity, this linkage will only be for the period of the research and not available to moderators or other forum users.

We will adhere to British Psychological Society guidance (43) for online informed consent including: taking a record of valid consent; check boxes relating to specific consent statements; limiting the number of consent items; and ensuring participants were fully informed of study procedures, risks, confidentiality and right to withdraw.

The survey design will be guided by the programme theories. Where established measures of hypothesised impacts on forum member emotional wellbeing or functioning are available, these will be used (e.g. GAD-7 for anxiety (44) PHQ-8 for mood (45) and where not, questions will be created.

Context will be measured at several levels and include any features of the forum, or backdrop against which it is being delivered, or the individual members, that may influence the extent to which hypothesised mechanisms are triggered. We will collect information on participant use of both NHS and privately funded healthcare services and medicines, in addition to community and third-sector services, related to their mental health. using an adapted online version of the Client Service Receipt Inventory (24) and other validated resource use questionnaires.

All forum users over 16 years old will be eligible to take part in the survey, and we can compare demographic and usage data of the sample of those who do participate in the survey with the whole forum sample (where this data is available).

For peer review only

Analysis of survey data will include detailed descriptions of the forum samples and summary statistics on mental health outcomes. We will use the Mplus (v8.6) software package (46, 47) to fit generalized mixed models and generalized structural equation models of the data, accounting for the hierarchal nature of the longitudinal data at the level of posts, users, and forums. We used simulation results (48) to estimate the sample size required to have statistical power of at least 0.8 to detect a small (a*b = 0.02) longitudinal mediation effect across three waves of data. Assuming an Intra-Class Correlation of 0.6, a significance level of p = 0.05, and 20% attrition per wave, we would need 602 participants, which is highly feasible across all forums.

Qualitative Interviews

We will interview key stakeholders online including approximately 10-12 forum members, 4-5 moderators, and 1-2 hosts or commissioners in each of our forum cases (total sample up to 114). This will enable us to test our hypotheses, whilst also being manageable within the resources of the project. Guided by realist interviewing methodology (49, 50), theoretical sampling for forum members will be determined using an evolving sampling framework, drawing on our survey and analysis of forum posts to identify which participants are needed to test our programme theories. We will sample across observers, super-users and regular forum members, as well as people who have been invited to join an online forum but not done so.

Interview topic guides will include an opportunity for participants to share what they understand the role of online forums to be, their impacts, and how they "work". With our moderators, host and commissioners we will also explore their understanding of the role of moderators, how they make decisions regarding moderating behaviours, and how they are trained and supported in this role. Interview topic guides will evolve to specifically test our hypotheses. Interviews will be face-to-face or via secure video conferencing (Microsoft Teams), or telephone, recorded and transcribed. Analysis will first be done within case, and then integrated to compare between cases. Data will be managed in NVIVO. Consistent with the realist approach, analysis will be retroductive i.e., seek to identify the hidden causal forces underlying people's descriptions of their experiences in the forums. These will be initially coded into our hypothesised CMO configurations from Workstream 1, but the analysis will be developed through regular discussion with the research team and PPI/Codesign group.

Analysis of forum posts

The collection of posts from our online forums will result in large linguistic datasets, potentially amounting to millions of words in some cases. To analyse these, we will use Corpus-based Discourse Analysis and Natural Language Processing (51). We will first describe the size, shape, content, emotional tone and linguistic features of each of our forum cases, also considering how each of these features change over time. In the second stage, we will adapt methods to test our specific CMO configurations. Specific techniques we can draw on to do this are summarised in Table 1. Analyses will be carried out using the latest versions of cutting-edge corpus tools developed at Lancaster University (#LancsBox, the Lancaster Stats Tools Online, lexiDB and Wmatrix), and, where appropriate, the corpus analysis tools available in AntLab, and Sketch Engine and state-of-the-art NLP libraries and repositories such as Spacy and Hugging Face.

Table 1- Overview of computational linguistic techniques to analyse forum posts

Stage 1 technique	Function
Frequency and dispersion analyses	To obtain the frequencies and distribution of selected words, phrases or semantic domains in a corpus and its sub-sections.

For peer review only

Keyness analyses	To identify and study the words, phrases and concepts characteristic of a particular (sub-)corpus, e.g.: an online community vs. a general corpus of English; moderated vs. unmoderated communities; first posts vs. last posts; patients' posts vs. moderators' posts; etc.
Change over time analyses	To track changes in co-occurring words of critical interest over time to capture continuities and discontinuities in meanings and associations
Summarisation or topic modelling	To generate short summaries of forums or parts of forums and locate the most important posts.
Social network analysis	To extract the structure and shape of the network of people in the community, to see who is talking to whom, to classify user's participation style (observers, users, super-users).
User profiling	To estimate the relative usage and representativeness of people in each community by age and gender, country location and other demographic variables
**Stage 2 technique**	**Function**
Concordance analyses	To obtain all occurrences of selected words, phrases or instances of different semantic domains, and to understand how and why they are used, e.g.: words such as 'feel', 'daughter', and 'medication' or semantic domains such as 'Sad', 'Personal relationships' and 'Medicines and medical treatment'.
Collocation analyses	To study patterns of co-occurrence of words or phrases, which are well known to contribute to their meanings and associations, e.g. what words tend to co-occur with 'you', 'not' or 'worry' in different (sub-)corpora.
Sentiment analysis	To study change in emotion over time and across forums or threads, showing positive and negative emotions, and we will combine this with an analysis of risk indicator words and taboo words, and terms of emotional support or bullying.

Qualitative analyses will be employed to carry out in-depth studies of specific posts, interactions or threads selected to test our specific theories. This might include threads with particularly high or low frequencies of negatively valanced emotion words; posts or threads that were found to be prototypical of a whole forum in linguistic terms; posts that received large numbers of replies; posts by individuals representing different levels of engagement or roles with the online forum, or showing evidence of significant change in emotional state. The qualitative analyses will investigate how users and moderators express and interactively manage identities, experiences, knowledge, emotions and relationships, and with what local impacts in terms of patterns in responses and interactional behaviour. These analyses will be directed at testing our CMOs by exploring users' understandings of mechanisms underlying the impact that participation in the online forum has on themselves and others.

Triangulation of data across methods and between cases

All methods have limitations, and in combining our survey, interviews, and analysis of forum posts, we can ensure that: we have a full range of perspectives (including those who choose not to take part in all aspects); we can purposively sample for interview on the basis of self-reported and real time evidence of specific impacts, or membership to specific subgroups; we can move between broad contextual data, such as the size and structure of the forum, to detailed analysis of individual conversations: we can test specific programme theories in multiple ways to test reliability and validity of findings. Regular analysis meetings, including our PPI/Codesign group, will ensure that we

For peer review only

make the most of the opportunity for iterative and integrated analysis across our cases and between our researchers using different methods.

Workstream 3 (Months 2-34) Impact

Our theories will be used to underpin the co-design of best-practice tools and support for people involved in commissioning, hosting, moderating or using online mental health forums (**RQ3)**. All tools will be informed by the programme theory developed in Workstreams 1 & 2. The exact nature of the tools will depend on the outcomes of the co-design process, but our pre-application work with our PPI, hosts and impact groups has suggested the candidate tools described in Table 2.

We will design all outputs and our dissemination strategy to be accessible across age, gender, ethnicity, disability and the digital divide. We will write plain English summaries, translated into other languages where possible, and disseminate via non-digital platforms (in person, radio, & print media) as well as digitally (websites, social media etc).

Table 2. Candidate tools and implementation

Tool	Aim
For people with mental health difficulties, referrers, and commissioners	
Knowledge exchange video/animation tools	Widening access and promoting greater uptake through better understanding of the role of online mental health forums and how they work. These tools will describe how online forums work, possible benefits, for who, and how to identity safe and supportive forums.
For forum moderators	
Community of Practice (CoP)	Building capacity through facilitating mutual engagement, joint enterprise, and shared practice. Such support is crucial to continuing professional development, and to prevent compassion fatigue and burnout. Openly available to all the moderators involved in our study and providing a safe online space for the forum to evolve from the outset of the study.
E-learning curricula	To train and support moderators in reflexive practice including: understanding the moderator role; ethics of moderation; encouraging activity; understanding mental health; spotting moments of change (introductory linguistic analysis); managing challenging situations; widening access and welcoming diversity; identifying and managing risks; looking after yourself; role of supervision & peer support; signposting; continuing professional development

For peer review only

	(CPD). The content will draw on challenges described by moderators during our interviews, and practical case examples of ways to manage these.
For forum providers/hosts, commissioners and policy makers	
Best practice design principles for online mental health forums	To widen access, maximise positive impacts, and minimise harms. This guidance will provide 1) High level design principles to guide forum design and moderation. 2) Practical guidance for implementing design features to improve usefulness and safety of online forums 3) Examples of best practice and case studies on forum design and moderation practices.
Resource requirements for implementation of best practice design principles based on the disaggregation of the programme theories into resources and reasoning	This will be based on the disaggregation of the programme theories into resources and reasoning and will provide information on the resource configuration necessary to provide safe and effective online community.
Standardised forum characterisation and evaluation framework	To support standardised evaluation and comparison of forums during commissioning
Methodological guidance on how to evaluate online mental health forums	To provide guidance for evaluation of efficacy and effectiveness
For further theory and practice development	
Programme theory	To generate a theory that is generalisable to online mental health forums and with relevance to understanding online peer support interventions across healthcare.

Knowledge Mobilisation

As the host site for this study, Berkshire Healthcare NHS Foundation Trust will take a lead role in ensuring the best practice tools outputs will be easily discovered by our relevant audiences, openly accessible, free to use, and updated and maintained beyond the life of the project. Ongoing feedback and revisions will be built into the design to ensure these tools evolve as needed. We will also work with the Health Education England learning hub (https://learninghub.nhs.uk), NHS England, ORCHA (https://orchahealth.com) and Oxford AHSN to explore how these tools can be best designed and promoted across moderators working across NHS, charity and commercial organisations.

Discussion

Online mental health forums have the potential to greatly increase access to peer support, but the impacts of using forums, and the underlying mechanisms, need to be better understood. This protocol outlines a methodologically novel triangulation of survey, interview and corpus linguistic methods, to generate and test realist programme theories about how online forums "work" for forum users and moderators, and to use these theories to develop best policy and practice tools to improve peer online forums. This study will be an inherently iterative process, and therefore the aim

For peer review only

of the protocol is not to prospectively define all our decisions at the outset, but rather to explore some of the key issues that have arisen and to share our learning from the challenges we have faced in designing the protocol.

The first challenge was to develop an ethical framework for the use of online forums posts as research data. We adopted a user centred approach, considering a user's likely expectations and concerns; consulted health forum hosts, moderators and users; hosted an online focus group (N = 21) exploring the study's benefits and risks and how best to mitigate these; examined key guidance on using online data including a) the British Psychological Society (43) and b) Association of internet researchers (52); collaborated with legal experts at Berkshire Healthcare NHS Foundation Trust and the information officer at Lancaster University, and discussed our strategy widely to learn how other researchers in the field have managed these ethical issues. The outcome of this process is described in our Data Processing Plan shown in Table 3 below and published on our study website (www.lancaster.ac.uk/ipof/ethics-framework). The process raised several interesting ethical issues which will be described in detail in a separate paper.

The second challenge was to build a team of collaborators, each expert in their own discipline, but not all familiar with the realist approach. Statistical analysis of surveys, reflexive thematic analysis of interview transcripts, corpus linguistic analysis of forum posts, and realist synthesis of published literature, all originate from different philosophical and theoretical positions, and vary in the extent to which they use different styles of reasoning (inductive, deductive, abductive, retroductive etc.). We have divided the project into workstreams which focus initially on the set up and descriptive analysis of the survey, interview and linguistic data. However, we have also built a collaborative workstream throughout the project to focus on developing a shared understanding of the realist approach, to codevelop the programme theories, and codesign novel integrated methods to test these.

The final challenge has been establishing our PPI/Codesign group. We wanted to ensure moderators and forum members for all our participating forums had a significant ongoing role throughout the project. They have the expertise to ensure the study is designed and conducted in a way that is engaging and acceptable to participants (PPI work), and the outputs are designed to be useful and effective (codesign participation). We have planned for this group to meet monthly throughout the study. However, despite increasingly detailed guidance from the NIHR about payment for involvement (53), this has been a challenge. Due to the extent and longevity of involvement, PPI members are required to be employees of the University, to share evidence of their right to work in the UK and navigate a bureaucratic contract-based payment process. These payment methods were not designed for the inherently flexible and iterative nature of PPI co-design work described above. Fear of losing access to benefits due to being deemed able to work as a result of getting involved in this kind of project has been another barrier to engagement. This can exclude those from more marginalised groups, whose voices need to be heard in this kind of research.

Ethics

Ethical approval was granted by Solihull Research Ethics Committee (**IRAS 314029**) on the 20th of June 2022. Table 3 describes each step in the process from data collection to dissemination for each of the three different types of data being collected.

Table 3 – Data Processing Plan for iPOF.

	Forum Data	Survey Data	Interview Data

For peer review only

**Collection**	Posts from publicly available forums with no expectation of privacy, where the forum host consents, will be accessed directly. Prior to downloading the data, the study will be described and users invited to withdraw their data if they wish.<br>Posts from forums requiring a login, with an expectation of privacy, will only be used from individuals who have freely consented (i.e. they had the option to not consent and still use the service) to their data being used for research. Prior to downloading the data, the study will be described, and users invited to have their data removed if they wish.<br>Posts from forums in which consent for data to be used for research is required before joining the forum, will be used, but only where it is possible to ensure all users have been made aware of the option to opt out.<br>Posts from forums linked to health or social care records, and no consent has been given for research at sign up, will only be used with additional individual informed consent.<br>Finally, posts from forums that are closed, publicly available, and where there are no links between posts and any personal identifiable data will be used without additional consent.	Using REDCap with individual informed consent.	Using Microsoft Teams and an encrypted audio recorder, with individual informed consent
**De-Identification**	When forums are anonymous and publicly available, we will replace all usernames with a PIN and automatically remove any names of places or people that could be identifiable.<br>For all other forums, posts will be deidentified by the host organisation before being shared with us.	Identifiable data, such as on consent forms, will be stored separately to the survey results.	Identifying information will be removed from transcripts. Identifiable data, such as on consent forms, will be stored separately.
**Transfer**	Data transfer from online communities to Lancaster University will be secure and encrypted (e.g. secure FTP and HTTPS).	Data will be collected via a link directly into Lancaster University system.	Audio files will be uploaded to Lancaster's servers and deleted from the recorder. Transcription will be done by a University approved and contracted transcriber.
**Storage**	Using Lancaster University's approved IT systems.	Data will be stored on Lancaster University's secure servers.	Recordings and transcripts will be stored on Lancaster University's secure servers.
**Analysis**	Analysis (Natural Language Processing) will be conducted by methodological expert members of the research team. (See Table 1)	Analyses will include detailed description of the sample and the use of generalized mixed models and structural	Analysis will be retroductive and will contribute to hypothesised CMO

For peer review only

		equation models using Mplus (v8.6).	configurations. Analysis will be managed in NVIVO.
**Deletion**	Participants will have the right to withdraw and request that their data be deleted, up to the analysis starting.	Participants have 1 week to request their data to be removed.	Participants will have the right to withdraw and request that their data be deleted, up to the analysis starting.
**Publication**	To preserve anonymity, paraphrased forum quotes will be published.	Minimum cell sizes will be adopted for published results.	If consent is given, direct quotes will be published. Any potentially identifying information will be removed.
**Archiving & Access**	All papers will be published open access. Given the nature of the data we will not share the forum datasets openly.	Deidentified survey data will be openly shared on PURE.	Interview data will be restricted access and available by request to legitimate research parties if the purpose is consistent with the consent given for this research.

For peer review only

Dissemination of findings

We will define our range of audiences, identify what each needs to know, and prepare targeted outputs appropriately created and written for this audience including Plain English summaries. Findings will be reported in accordance with RAMESES (1) guidelines, published as open access and shared widely, along with codesigned tools. Study outputs will be promoted through a national launch of the co-designed tools, as part of a conference, webinar, and social media strategy. Our protocols and academic findings will be published in open access peer reviewed journals and presented at academic and peer forum conferences. All study participants and relevant health, social, educational and academic organisations will be kept updated via a study website (https://www.lancaster.ac.uk/ipof), social media (twitter), and blogs from across the wider team. All policy and practice guidance will be hosted, updated, and made widely available via Berkshire Healthcare NHS Foundation Trust.

Authors contributions

All authors contributed to drafting the manuscript and approved the final version. The study was conceived by FL, developed for funding by FL, JRM, ES, NS, PR, SJ, CL, RM, SW, MR, and further adapted in the setup phase by input from all authors.

Theory development and testing is led by PM, HR, and MC, with guidance in realist methodology from JRM. Qualitative analysis of interviews and corpus linguistics is led by ES; computational analysis by PR and MC; survey design and analysis by NS; codesign by SJ, and KM; PPI by CL. The programme is managed by TR, with research administration support from RJ; ZG; KP, and ED. Clinical input is provided by SJ; FL; SW and JH.

References

1. The RAMESES Projects 2022 [Available from: https://www.ramesesproject.org/.
2. McManus S, Bebbington PE, Jenkins R, Brugha T. Mental Health and Wellbeing in England: the Adult Psychiatric Morbidity Survey 2014: NHS digital; 2016.
3. NHS Confederation. Digital inclusion in mental health - a guide to help increase choice and improve access to digital mental health services. 2020.

4. Ofcom. Children and parents: Media use and attitudes report 2019. Ofcom London, UK; 2020.
5. Saha K, Sharma A, editors. Causal factors of effective psychosocial outcomes in online mental health communities. Proceedings of the International AAAI Conference on Web and Social Media; 2020.
6. Hanley T, Prescott J, Gomez KU. A systematic review exploring how young people use online forums for support around mental health issues. Journal of Mental Health. 2019;28(5):566-76.
7. Naslund J, Aschbrenner K, Marsch L, Bartels S. The future of mental health care: peer-to-peer support and social media, Epidemiology and Psychiatric Sciences, 25, 113-122. 2016.
8. Coulson NS, Bullock E, Rodham K. Exploring the therapeutic affordances of self-harm online support communities: An online survey of members. JMIR mental health. 2017;4(4):e44.
9. Brady E, Segar J, Sanders C. "I always vet things": navigating privacy and the presentation of self on health discussion boards among individuals with long-term conditions. Journal of medical Internet research. 2016;18(10):e274.
10. Griffiths KM, Reynolds J, Vassallo S. An online, moderated peer-to-peer support bulletin board for depression: user-perceived advantages and disadvantages. JMIR Mental Health. 2015;2(2):e14.
11. Powell J, McCarthy N, Eysenbach G. Cross-sectional survey of users of Internet depression communities. BMC psychiatry. 2003;3(1):19.
12. Prescott J, Rathbone AL, Hanley T. Online mental health communities, self-efficacy and transition to further support. Mental Health Review Journal. 2020.
13. Bartlett YK, Coulson NS. An investigation into the empowerment effects of using online support groups and how this affects health professional/patient communication. Patient education and counseling. 2011;83(1):113-9.
14. Smith-Merry J, Goggin G, Campbell A, McKenzie K, Ridout B, Baylosis C. Social connection and online engagement: insights from interviews with users of a mental health online forum. JMIR mental health. 2019;6(3):e11084.
15. Griffiths KM, Mackinnon AJ, Crisp DA, Christensen H, Bennett K, Farrer L. The effectiveness of an online support group for members of the community with depression: a randomised controlled trial. PloS one. 2012;7(12):e53244.
16. Marchant A, Hawton K, Stewart A, Montgomery P, Singaravelu V, Lloyd K, et al. A systematic review of the relationship between internet use, self-harm and suicidal behaviour in young people: The good, the bad and the unknown. PloS one. 2017;12(8):e0181722.
17. Dunlop SM, More E, Romer D. Where do youth learn about suicides on the Internet, and what influence does this have on suicidal ideation? Journal of child psychology and psychiatry. 2011;52(10):1073-80.
18. Moore D, Ayers S. Virtual voices: social support and stigma in postnatal mental illness Internet forums. Psychology, health & medicine. 2017;22(5):546-51.
19. Rodgers RF, Lowy AS, Halperin DM, Franko DL. A meta-analysis examining the influence of pro-eating disorder websites on body image and eating pathology. European Eating Disorders Review. 2016;24(1):3-8.
20. Bailey E, Robinson J, Alvarez-Jimenez M, Nedeljkovic M, Valentine L, Bendall S, et al. Moderated online social therapy for young people with active suicidal ideation: qualitative study. Journal of medical internet research. 2021;23(4):e24260.
21. Easton K, Diggle J, Ruethi-Davis M, Holmes M, Byron-Parker D, Nuttall J, et al. Qualitative Exploration of the Potential for Adverse Events When Using an Online Peer Support Network for Mental Health: Cross-Sectional Survey. JMIR mental health. 2017;4(4):e49.
22. Ziebland S, Wyke S. Health and illness in a connected world: how might sharing experiences on the internet affect people's health? The Milbank Quarterly. 2012;90(2):219-49.

For peer review only

23. Malik S, Coulson NS. 'They all supported me but I felt like I suddenly didn't belong anymore': an exploration of perceived disadvantages to online support seeking. Journal of Psychosomatic Obstetrics & Gynecology. 2010;31(3):140-9.
24. Lobban F, Appelbe D, Appleton V, Billsborough J, Fisher NR, Foster S, et al. IMPlementation of An online Relatives' Toolkit for psychosis or bipolar (IMPART study): iterative multiple case study to identify key factors impacting on staff uptake and use. BMC health services research. 2020;20(1):1-13.
25. Lobban F, Appelbe D, Appleton V, Aref-Adib G, Barraclough J, Billsborough J, et al. An online supported self-management toolkit for relatives of people with psychosis or bipolar experiences: the IMPART multiple case study. Health Services and Delivery Research. 2020;8(37).
26. Krasodomski-Jones A. Everything in moderation: platforms, communities and users in a healthy online environment.
27. Sibilla F, Mancini T. I am (not) my avatar: A review of the user-avatar relationships in Massively Multiplayer Online Worlds. Cyberpsychology: Journal of Psychosocial Research on Cyberspace. 2018;12(3).
28. Suler J. The online disinhibition effect. Cyberpsychology & behavior. 2004;7(3):321-6.
29. SMITH J, KRASODOMSKI-JONES A, OLANIPEKUN M, JUDSON E. A picture of health march 2021: Measuring the comparative health of online spaces. 2021.
30. Grimmelmann J. The virtues of moderation. Yale JL & Tech. 2015;17:42.
31. Smith J, Jones E, Judson E. What's in a name? A forward view of anonymity online.
32. Yin RK. Case study research and applications: Design and methods: Sage publications; 2017.
33. Staniszewska S, Brett J, Simera I, Seers K, Mockford C, Goodlad S, et al. GRIPP2 reporting checklists: tools to improve reporting of patient and public involvement in research. bmj. 2017;358.
34. Pawson R, Greenhalgh T, Harvey G, Walshe K. Realist synthesis: an introduction. Manchester: ESRC Research Methods Programme, University of Manchester. 2004.
35. Rycroft-Malone J, McCormack B, Hutchinson AM, DeCorby K, Bucknall TK, Kent B, et al. Realist synthesis: illustrating the method for implementation research. Implementation Science. 2012;7(1):33.
36. Jagosh J. Realist synthesis for public health: building an ontologically deep understanding of how programs work, for whom, and in which contexts. Annual review of public health. 2019.
37. Hunter R, Gorely T, Beattie M, Harris K. Realist review. International Review of Sport and Exercise Psychology. 2022;15(1):242-65.
38. Wadden D, August T, Li Q, Althoff T. The Effect of Moderation on Online Mental Health Conversations. arXiv preprint arXiv:200509225. 2020.
39. Bonell C, Jamal F, Melendez-Torres G, Cummins S. 'Dark logic': theorising the harmful consequences of public health interventions. J Epidemiol Community Health. 2015;69(1):95-8.
40. Kaylor-Hughes CJ, Rawsthorne M, Coulson NS, Simpson S, Simons L, Guo B, et al. Direct to public peer support and e-therapy program versus information to aid self-management of depression and anxiety: protocol for a randomized controlled trial. JMIR research protocols. 2017;6(12):e231.
41. Powell J, Hamborg T, Stallard N, Burls A, McSorley J, Bennett K, et al. Effectiveness of a web-based cognitive-behavioral tool to improve mental well-being in the general population: randomized controlled trial. Journal of medical Internet research. 2013;15(1):e2.
42. Patridge E, Bardyn T. Research Electronic Data Captrue (REDCap). Journal of the Medical Library Association. 2018;106(1):142-4.
43. Hewson C, Buchanan T, editors. Ethics guidelines for internet-mediated research2013: The British Psychological Society.
44. Spitzer RL, Kroenke K, Williams JB, Löwe B. A brief measure for assessing generalized anxiety disorder: the GAD-7. Archives of internal medicine. 2006;166(10):1092-7.
45. Kroenke K, Spitzer RL, Williams JB. The PHQ-9: validity of a brief depression severity measure. Journal of general internal medicine. 2001;16(9):606-13.

46. Maxwell SE, Cole DA. Bias in cross-sectional analyses of longitudinal mediation. Psychological methods. 2007;12(1):23.
47. Rabe-Hesketh S, Skrondal A, Pickles A. Generalized multilevel structural equation modeling. Psychometrika. 2004;69(2):167-90.
48. Pan H, Liu S, Miao D, Yuan Y. Sample size determination for mediation analysis of longitudinal data. BMC Medical Research Methodology. 2018;18(1):1-11.
49. Pawson R. Theorizing the interview. British Journal of Sociology. 1996:295-314.
50. Manzano A. The craft of interviewing in realist evaluation. Evaluation. 2016;22(3):342-60.
51. Baker P, Gabrielatos C, Khosravinik M, Krzyżanowski M, McEnery T, Wodak R. A useful methodological synergy? Combining critical discourse analysis and corpus linguistics to examine discourses of refugees and asylum seekers in the UK press. Discourse & society. 2008;19(3):273-306.
52. Research AoI. Ethics: Association of Internet Research; [Available from: https://aoir.org/ethics/.
53. NIHR. Payment guidance for researchers and professionals. NIHR. 2021.

For peer review only

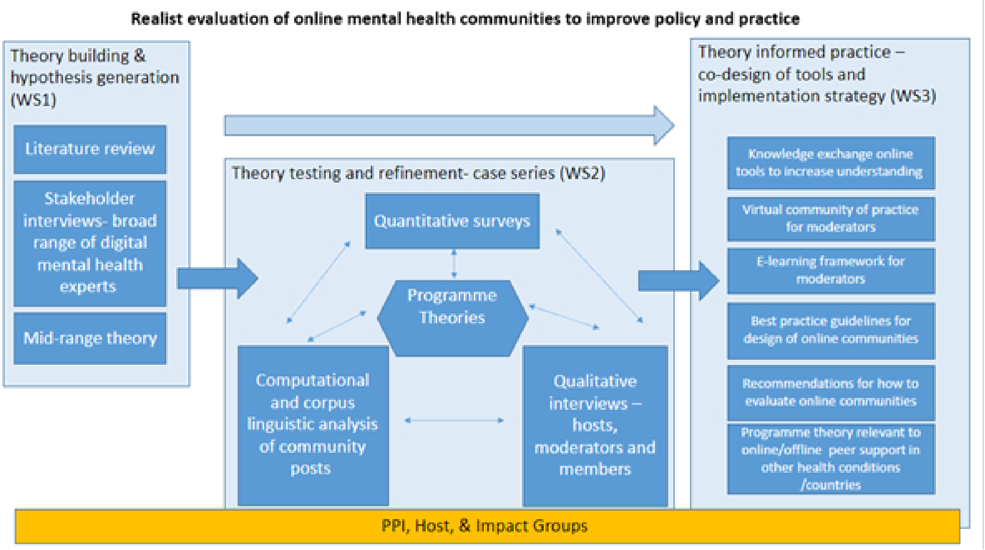

402x221mm (38 x 38 DPI)

BMC Medicine

Table 1 List of items to be included when reporting a realist synthesis

From: RAMESES publication standards: realist syntheses

TITLE		
1		In the title, identify the document as a realist synthesis or review
ABSTRACT		
2		While acknowledging publication requirements and house style, abstracts should ideally contain brief details of: the study's background, review question or objectives; search strategy; methods of selection, appraisal, analysis and synthesis of sources; main results; and implications for practice.
INTRODUCTION		
3	Rationale for review	Explain why the review is needed and what it is likely to contribute to existing understanding of the topic area.
4	Objectives and focus of review	State the objective(s) of the review and/or the review question(s). Define and provide a rationale for the focus of the review.
METHODS		
5	Changes in the review process	Any changes made to the review process that was initially planned should be briefly described and justified.
6	Rationale for using realist synthesis	Explain why realist synthesis was considered the most appropriate method to use.
7	Scoping the literature	Describe and justify the initial process of exploratory scoping of the literature.
8	Searching processes	While considering specific requirements of the journal or other publication outlet, state and provide a rationale for how the iterative searching was done. Provide details on all the sources accessed for information in the review. Where searching in electronic databases has taken place, the details should include, for example, name of database, search terms, dates of coverage and date last searched. If individuals familiar with the relevant literature and/or topic area were contacted, indicate how they were identified and selected.

For peer review only - http://bmjopen.bmj.com/site/about/guidelines.xhtml

TITLE		
9	Selection and appraisal of documents	Explain how judgements were made about including and excluding data from documents, and justify these.
10	Data extraction	Describe and explain which data or information were extracted from the included documents and justify this selection.
11	Analysis and synthesis processes	Describe the analysis and synthesis processes in detail. This section should include information on the constructs analyzed and describe the analytic process.
RESULTS		
12	Document flow diagram	Provide details on the number of documents assessed for eligibility and included in the review with reasons for exclusion at each stage as well as an indication of their source of origin (for example, from searching databases, reference lists and so on). You may consider using the example templates (which are likely to need modification to suit the data) that are provided.
13	Document characteristics	Provide information on the characteristics of the documents included in the review.
14	Main findings	Present the key findings with a specific focus on theory building and testing.
DISCUSSION		
15	Summary of findings	Summarize the main findings, taking into account the review's objective(s), research question(s), focus and intended audience(s).
16	Strengths, limitations and future research directions	Discuss both the strengths of the review and its limitations. These should include (but need not be restricted to) (a) consideration of all the steps in the review process and (b) comment on the overall strength of evidence supporting the explanatory insights which emerged.<br><br>The limitations identified may point to areas where further work is needed.
17	Comparison with existing literature	Where applicable, compare and contrast the review's findings with the existing literature (for example, other reviews) on the same topic.
18	Conclusion and recommendations	List the main implications of the findings and place these in the context of other relevant literature. If appropriate, offer recommendations for policy and practice.
19	Funding	Provide details of funding source (if any) for the review, the role played by the funder (if any) and any conflicts of interests of the reviewers.

Back to article page ›

BMC Medicine

ISSN: 1741-7015

Contact us

Submission enquiries: bmcmedicineeditorial@biomedcentral.com

For peer review only - http://bmjopen.bmj.com/site/about/guidelines.xhtml

BMJ Open

Improving Peer Online Forums (iPOF): Protocol for a Realist Evaluation of Peer Online Mental Health Forums to Inform Practice and Policy

Journal:	*BMJ Open*
Manuscript ID	bmjopen-2023-075142.R1
Article Type:	Protocol
Date Submitted by the Author:	12-Jul-2023

Complete List of Authors:	Lobban, Fiona; University of Lancaster, Division of Health Research<br>Coole, Matthew; Lancaster University<br>Donaldson, Emma; Berkshire Healthcare NHS Foundation Trust<br>Glossop, Zoe; Lancaster University, Spectrum Centre for Mental Health Research<br>Haines, Jade; Berkshire Healthcare NHS Foundation Trust<br>Johnston, Rose; Lancaster University, Spectrum Centre for Mental Health Research<br>Jones, Steven; Lancaster University, Spectrum Centre for Mental Health Research, Division of Health Research, Faculty of Health and Medicine<br>Lodge, Christopher; Lancaster University, Spectrum Centre for Mental Health Research<br>Machin, Karen; Independent Survivor Researcher<br>Marshall, Paul; Lancaster University, Spectrum Centre for Mental Health Research, Division of Health Research<br>Meacock, Rachel; University of Manchester<br>Penhaligon, Kate; Berkshire Healthcare NHS Foundation Trust<br>Rakić, Tamara; Lancaster University<br>Rawsthorne, Mat; Service User Researcher<br>Rayson, Paul; Lancaster University<br>Robinson, Heather; Lancaster University<br>Rycroft-Malone, Jo; Lancaster University<br>Semino, Elena; Lancaster University, Linguistics and English Language<br>Shryane, Nick; The University of Manchester School of Social Sciences, Social Statistics<br>Wise, Sara; Berkshire Healthcare NHS Foundation Trust

**Primary Subject Heading**:	Mental health
Secondary Subject Heading:	Mental health, Research methods
Keywords:	MENTAL HEALTH, Patient Participation, QUALITATIVE RESEARCH, HEALTH ECONOMICS

Improving Peer Online Forums (iPOF): Protocol for a Realist Evaluation of Peer Online Mental Health Forums to Inform Practice and Policy

Author Information

Fiona Lobban, Lancaster University, f.lobban@lancaster.ac.uk, ORCID: 0000-0001-6594-4350 (Corresponding Author)

Matthew Coole, Lancaster University, m.coole@lancaster.ac.uk, ORCID: 0000-0002-9279-931X

Emma Donaldson, Berkshire Healthcare NHS Foundation Trust, Emma.Donaldson@berkshire.nhs.uk, ORCID: 0000-0003-0304-8360

Zoe Glossop, Lancaster University, z.glossop@lancaster.ac.uk, ORCID: 0000-0002-6151-4860

Jade Haines, Berkshire Healthcare NHS Foundation Trust, jade.haines@berkshire.nhs.uk

Rose Johnston, Lancaster University, r.johnston2@lancaster.ac.uk, ORCID: 0000-0002-6863-2624

Steven Jones, Lancaster University, s.jones7@lancaster.ac.uk, ORCID: 0000-0002-8801-5113

Christopher Lodge, Lancaster University, c.lodge@lancaster.ac.uk, ORCID: 0000-0003-1115-5553.

Karen Machin, Independent survivor researcher, karen@role.org.uk, ORCID 0000-0002-0374-4238

Paul Marshall, Lancaster University, p.marshall4@lancaster.ac.uk, ORCID: 0000-0002-3913-3359

Rachel Meacock, The University of Manchester, rachel.meacock@manchester.ac.uk, ORCID: 0000-0001-8933-5058

Kate Penhaligon, Berkshire Healthcare NHS Foundation Trust, kate.penhaligon@berkshire.nhs.uk

Tamara Rakić, Lancaster University, t.rakic@lancaster.ac.uk, ORCID: 0000-0002-8591-923X

Mat Rawsthorne, Service User Researcher, mat.rawsthorne@proton.me, ORCID 0000-0002-7481-693X

Paul Rayson, UCREL, School of Computing and Communications, Lancaster University, p.rayson@lancaster.ac.uk, ORCID: 0000-0002-1257-2191

Heather Robinson, Lancaster University, h.robinson@lancaster.ac.uk, ORCID: 0000-0002-3566-2253

Jo Rycroft-Malone, Faculty of Health & Medicine, Lancaster University, j.rycroft-malone1@lancaster.ac.uk, ORCID: 0000-0003-3858-5625

Elena Semino, Department of Linguistics and English Language, and ESRC Centre for Corpus Approaches to social Science, Lancaster University, e.semino@lancaster.ac.uk, ORCID: 0000-0002-3421-2963

Nick Shryane, Department of Social Statistics, University of Manchester, N.Shryane@manchester.ac.uk, ORCID 0000-0001-7963-4601

For peer review only

Sara Wise, Berkshire Healthcare NHS Foundation Trust, sara.wise@berkshire.nhs.uk

Word Count

4843

For peer review only

Abstract

Introduction. Peer online mental health forums are commonly used and offer accessible support. Positive and negative impacts have been reported by forum members and moderators, but it is unclear why these impacts occur, for whom, and in which forums. This multiple method realist study explores underlying mechanisms to understand how forums work for different people. The findings will inform codesign of best practice guidance and policy tools to enhance the uptake and effectiveness of peer online mental health forums.

Methods and Analysis. In workstream 1 we will conduct a realist synthesis, based on existing literature and interviews with approximately 20 stakeholders, to generate initial programme theories about the impacts of forums on members and moderators and mechanisms driving these. Initial theories that are relevant for forum design and implementation will be prioritised for testing in workstream 2.

Workstream 2 is a multiple case study design with mixed methods with several online mental health forums differing in contextual features. Quantitative surveys of forum members, qualitative interviews, and Corpus-based Discourse Analysis and Natural Language Processing of forum posts will be used to test and refine programme theories. Final programme theories will be developed through novel triangulation of the data.

Workstream 3 will run alongside workstream 1 and 2. Key stakeholders from participating forums, including members and moderators, will be recruited to a Codesign group. They will inform the study design and materials, refine and prioritise theories, and codesign best policy and practice guidance.

Ethics and dissemination. Ethical approval was granted by Solihull Research Ethics Committee (**IRAS 314029**). Findings will be reported in accordance with RAMESES guidelines, published as open access and shared widely, along with codesigned tools.

Registration details. The study is prospectively registered at ISRCTN 62469166. The protocol for the realist synthesis in workstream one is prospectively registered at PROSPERO CRD42022352528.

Strengths and limitations

- A detailed methodology is presented to develop and test a programme theory about how and why online forums can support people with mental health challenges and their carers. This methodology may inform the design of future studies to evaluate complex online interventions which are already in widespread use.
- A novel triangulation of survey, interview and corpus linguistic methods is described, that integrates the analysis of primary and secondary data sources, requiring collaborative working between interdisciplinary researchers with varying levels of experience in realist approaches.
- The process by which realist programme theory can be used to inform the codesign of best policy and practice guidance with people with lived expertise is outlined. This demonstrates the role of Patient and Public Involvement (PPI) in bridging the gap between theory development and intervention design.
- Ethical issues of using online forum posts in research are identified, and a framework for addressing these will be produced.

For peer review only

Introduction

Mental health problems affect approximately 1 in 6 people in the UK at any one time (>11million people) (1). People seek support in a variety of ways, increasingly through peer support and by going online (2, 3). They search for information and join online mental health forums: dedicated support platforms aimed at helping individuals to share, discuss and solicit information and support (4). Many are moderated, and some have access to additional online tools and resources. Although widely used, the impacts of using online forums, and mechanisms by which these occur, are poorly understood.

Online forums can provide round-the-clock opportunities for members to feel understood, access information, make friends, and use their experiences to help others (5) whilst being able to manage how they present themselves online (6-8). These forums can function as an important gateway to other offline support as people test out sharing their problems and are encouraged to access help (9-12). Online forums are particularly valued by people with rare or highly stigmatised difficulties, and those living in remote areas, or close knit communities wanting anonymity (13). Online forums offer robust support during pandemics when healthcare services are restricted, and for people in low- and middle-income countries where access to mobile technology is common but healthcare is not. Studies have shown positive outcomes associated with using online forums, including reduction in depression (14), suicidal thoughts (15), and social isolation (13).

However, negative impacts of using online forums have also been reported including increases in suicidal ideation (16), more negative mood (17), and worsening of body image and disordered eating patterns (18). Forums carry inherent risks of disinformation, dependency, and sharing of unhelpful strategies. Information shared can be misleading, inaccurate, harmful, or triggering(5, 19). Hearing about the experiences of others can exacerbate low mood and risk of harms (20) and generate unrealistic expectations and greater anxiety or confusion about one's own condition (21). Normative ideas about health may, leave some users feeling misunderstood or even bullied, and people in mental distress may be particularly vulnerable to the negative impacts of this kind of behaviour (22). Healthcare staff may be reluctant to moderate online forums because of concerns about possible harms and lack of protocols for online working (23), and may be reluctant to recommend specific forums without clearer guidance on criteria by which to make this decision (24).

Current research suggests a wide range of impacts from using online mental health forums, but with little understanding of why these vary across individuals (13-19). Particular features of forums such as size and heterogeneity, target population, ease of access and level of friction, how individual identities are managed, forum rules, and incentives for use and for pro-social behaviour have all been hypothesised as features that might account for the variation in impacts (25-28). Style of moderation is also likely to be important. The moderator role is highly variable across forums, ranging from identifying and removing risk-related materials, to that of a highly skilled online counsellor, directing the focus and values of the online forum (29).

Understanding how these contextual features influence the culture and function of forums is crucial. Hence, research needs to go beyond the study of behaviour in individual forums and investigate what aspects of design, moderation, and culture differ between forums, which facilitate better outcomes, and how (25, 30). Research to date has drawn on quantitative surveys, qualitative interviews, computational discourse analysis, and social network analysis, but all have limitations when used in isolation. Our research will move the field from single forum and single methods studies, to the triangulation of findings from a range of methods and online forums, sampled for contextual diversity, to test comprehensive programme theories. The programme theories will then be used to inform co-design of best practice and policy guidance with the aim of maximising positive impacts of online forums, whilst minimising risks of any negative impacts on users and moderators.

For peer review only

Aims and Objectives

Aims

To develop a programme theory to explain the underlying mechanisms by which online mental health forums impact on people's mental health and wellbeing.
To use this programme theory to develop best practice tools to improve uptake, safety and usefulness of online forums.

Objectives

Objectives are aligned to specific research questions (RQ).

RQ1: What are the impacts of using online mental health forums for people experiencing mental health difficulties? How, why, in what contexts, and for whom are these impacts generated?
Objectives
1a. Develop theories of the underlying generative mechanisms by which, and contexts within which, online mental health forums impact on mental health and wellbeing outcomes for members.
1b. Test and refine the theories in case studies of online mental health forums.

RQ2. What are the roles of forum moderators and how do they impact online mental health forums?
Objectives
2a. Develop a theory to understand the roles of online mental health forum moderators, how they vary in different contexts, and how they impact forums.
2b. Test and refine the theories in case studies of online mental health forums.

RQ3. How can evidence-based theories of online mental health forums be used to inform best practice guidance and support?
Objective
3a. Co-produce best practice tools, training and implementation plan to optimise the design and delivery of online mental health forums.

Methods and Analysis

Study design

A realist informed mixed methods evaluation, including multiple case studies (31) to co-design theory informed best practice guidance tools for online mental health forums.
Date collection will start in March 2022 and finish in December 2024

Figure 1 provides an overview of the study design.

[Figure 1. iPOF Study Design here]

Setting

Our research takes place online. Our cases are mental health forums, defined as:
online spaces to facilitate peer to peer sharing, which are aimed at young people and adults in the UK, and set up by a UK-based healthcare provider, charity, or commercial organisation with the explicit purpose of facilitating peer to peer support for people with mental health difficulties. Although people talk about mental health online in a variety of online spaces (including Facebook, Twitter, Instagram etc.), we have focussed on UK-based forums set up specifically to support mental

For peer review only

health, because we hypothesise that the impacts of forums will be strongly influenced by their broader social context, including access to other health and social care services.

We aim to recruit 6-8 forums, purposively sampled for diversity across host, target population, design (including level and nature of moderation, and whether they require registered login), and size of population. Forums will be identified using snowballing techniques and online searching. We aim for maximum variation in our sample.

Patient and public involvement (PPI) statement

Pre-funding, we hosted two online PPI events, each with twelve people with lived experience of moderating and/or regularly using online forums. Moderators reported significant challenges in their roles, impacting on their own mental health, and were very keen to receive better training and support. Forum members identified the need for healthcare staff and commissioners to be more aware of the role online forums can play in mental health support, and the need to understand the role of "super-users" who post a lot, and "observers" who read but do not post.

Our project management team includes people with lived experience of using online resources to manage mental difficulties guiding the design and delivery of the project.

We will establish a PPI/Codesign group consisting of forum members and moderators from participating forums. We will recruit widely across age, gender, ethnicity, disability, and digital engagement. The group will meet online monthly, facilitated by our PPI leads, and will contribute to the design and materials for the study, prioritise and refine programme theories, and codesign key outputs.

We also have two people with lived experience of using online mental health forums as independent members of our Study Steering Committee, meeting approximately every 6 months, to oversee the conduct of the study on behalf of the funder.

All PPI members will be invited to whole team training at the launch of the study to support a working understanding of all research methods drawn on in this study. Individual needs for training and support will be regularly reviewed and provided from across the team.

PPI will be reported in the final paper according to the GRIPP2 reporting checklist (32).

Methods

The study is organised in three parallel workstreams.

Workstream 1 (1-18 months). Theory building and hypothesis generation

Following established realist methodology (33-35), we will develop our initial candidate theories of how online mental health forums "work" (**RQ1a**) by drawing on well-established theories (36) reviewing relevant literature (including grey literature and intervention protocols), and conducting in-depth interviews with key stakeholders. The importance of forum moderation on outcomes is well established (5, 13, 29, 37), but not well understood. Therefore, to answer **RQ2a** we include a nested review question focussing specifically on understanding the role of the moderator and how this works in different contexts.

We will use the framing of If-then Context-Mechanism-Outcomes (CMO) configurations to guide all stages of the process, from theory construction to the development of data collection protocols and

For peer review only

data analysis. Outcomes will include positive and negative impacts on forum member emotional wellbeing or functioning, such as anxiety, mood, personal recovery, quality of life, and help seeking. Mechanisms will be defined as the responses of the forum members to the forum resources (including moderation) such as safety, toxicity, empathy, normalisation, bullying or reframing. Context will be explored at several levels and include any features of the forum, or backdrop against which it is being delivered, or the individual members, that may influence these mechanisms e.g. hosting, design, size, target population, or anonymity.

We will first develop an initial framework for candidate CMOs based on existing formal theories. Selection will be guided by the extent to which each theory addresses our research aims, and their compatibility with the realist approach and underlying philosophy i.e. to what extent they help to identify generative causal explanations for key outcomes.
Next, with additional support from our information specialists at Lancaster University Library, we will develop a strategy to review existing literature in relevant electronic databases such as Medline, PsychInfo, EMBASE etc., and additional grey literature (using Google, TRIP, overton.io, ProQuest, Allcatsrgrey, the International Clinical Trials Registry, and the NHS Knowledge and Library Hub) that report on the contexts in which online mental health forums are offered; the intended / proposed / measured outcomes; and explicit / implicit theories about how they "work". The search strategy will be systematic and clearly documented but will develop iteratively to include more diverse data sources that can shed light on generative causal pathways to outcomes. Data sources will include primary data, reviews, policy documents, training manuals, theoretical commentaries, author interpretations, websites and unpublished reports that give a rationale for offering online mental health forums, describe or evaluate online mental health forums, or detail the lived experiences of members/moderators/hosts/commissioners. Screening (initially by title and abstract, with 10% checked by a second reviewer) will be based on functional criteria of relevance, rigour and richness i.e. whether the document tells us anything about contexts, mechanisms, or outcomes of interest, rather than methodological quality (33). Data extraction will focus on extracting explanatory accounts and mapping where this data is coming from (the context, nature of the intervention etc.). In parallel to the literature review, we will interview up to 20 stakeholders including academics, clinicians, commissioners, policy makers, and moderators with broad expertise in online mental health forums. Sampling will be done through snowballing techniques, drawing on the networks of our broad research team, and key individuals identified in our literature searching. Data collection will be done flexibly (to maximise participation) using individual/ group interviews, online / face to face /telephone, and using topic guides designed to elicit CMO explanatory theories.

Analysis of literature and interview transcripts will be conducted using retroductive reasoning i.e. working back from the data to identify the context-dependent mechanisms underlying the impacts described in online forums. Particular attention will be paid to accounts of potential negative impacts ("dark logic models"(38)) and those that are unexpected or challenge our initial programme theories.

We will articulate a theory of how peer online forums impact forum members and moderators, starting with an array of potential outcomes, multiple possible mechanisms, and an initially broad context. We will work with our PPI/Codesign Group through prioritisation and consensus building, to ensure our final explanatory theory is comprehensible, testable, and useful for the design of policy and practice tools in WS3.

Workstream 2 (Month 6-30) Theory testing and refinement

In Workstream 2, hypotheses about 1) how online mental health forums work for whom, how and in what context (**RQ1b**), and 2) the role of forum moderators (**RQ2b**), will be tested in a longitudinal realist evaluation using novel triangulation of survey, interview, and corpus linguistic methods.

For peer review only

We will first develop a detailed case report for each forum describing key contextual features (based on aggregate data provided by the host) identified in Workstream 1 that influence user experiences e.g., design of forum (including access to additional online tools), ease of access and level of friction, size and heterogeneity of user population, how individual identities are managed (user identification vs anonymity), forum rules, and incentives for use and for pro-social behaviour. We will describe moderation strategies in each case including how moderators are recruited, trained and supported, moderating rules and how they are developed, temporality, transparency and opportunities for redress to moderation decisions. We will describe the linguistic properties of each forum in these case descriptions, focussing on the differences between the forums in terms of theme, topics of discussion, semantic categories, sentiment and emotion categories, and keywords.
Consistent with optimal case study design and realist approaches, we will take a mixed-method approach to explore relationships between quantitative measures of outcomes, contextual factors and some mediators, combined with an in-depth qualitative exploration of processes underpinning the deeper causal relationships between these variables. This will ensure that demi-regularities between variables across the sample are not in themselves assumed to be causal.

Surveys assessing impacts within and across cases
Surveys of forum users will quantitatively assess impacts of forum use, and evaluate potential mediators and moderators, at baseline, six weeks, and 12 weeks. The choice of these intervals is based on times to clinically important change observed in previous online peer-support studies (39, 40). Up to two iterations of the survey will allow for additional adaptation of measures should new hypotheses arise.

Participants will be recruited over a 9-month period across all forum cases, and will include members who passively view but rarely or never actively post to the forums (observers). Invitations and a link to the survey will be shared by forum hosts. On following that link, potential participants will see a participant information sheet (PIS), consent form, and only after providing their consent, the survey measures hosted on REDcap (41) to facilitate linkage between survey and forum use data participants will be invited to provide their username if they wish, and to indicate if they are willing to be contacted for an in-depth interview. To ensure user anonymity, this linkage will only be for the period of the research and not available to moderators or other forum users.
We will adhere to British Psychological Society guidance (42) for online informed consent including: taking a record of valid consent; check boxes relating to specific consent statements; limiting the number of consent items; and ensuring participants were fully informed of study procedures, risks, confidentiality and right to withdraw.
The survey design will be guided by the programme theories. Where established measures of hypothesised impacts on forum member emotional wellbeing or functioning are available, these will be used (e.g. GAD-7 for anxiety (43) PHQ-8 for mood (44) and where not, questions will be created.

Context will be measured at several levels and include any features of the forum, or backdrop against which it is being delivered, or the individual members, that may influence the extent to which hypothesised mechanisms are triggered. We will collect information on participant use of both NHS and privately funded healthcare services and medicines, in addition to community and third-sector services, related to their mental health. using an adapted online version of the Client Service Receipt Inventory (23) and other validated resource use questionnaires.
All forum users over 16 years old will be eligible to take part in the survey, and we can compare demographic and usage data of the sample of those who do participate in the survey with the whole forum sample (where this data is available).

For peer review only

Analysis of survey data will include detailed descriptions of the forum samples and summary statistics on mental health outcomes. We will use the Mplus (v8.6) software package (45, 46) to fit generalized mixed models and generalized structural equation models of the data, accounting for the hierarchal nature of the longitudinal data at the level of posts, users, and forums. We used simulation results (47) to estimate the sample size required to have statistical power of at least 0.8 to detect a small (a*b = 0.02) longitudinal mediation effect across three waves of data. Assuming an Intra-Class Correlation of 0.6, a significance level of p = 0.05, and 20% attrition per wave, we would need 602 participants, which is highly feasible across all forums.

Qualitative Interviews

We will interview key stakeholders online including approximately 10-12 forum members, 4-5 moderators, and 1-2 hosts or commissioners in each of our forum cases (total sample up to 114). This will enable us to test our hypotheses, whilst also being manageable within the resources of the project. Guided by realist interviewing methodology (48, 49), theoretical sampling for forum members will be determined using an evolving sampling framework, drawing on our survey and analysis of forum posts to identify which participants are needed to test our programme theories. We will sample across observers, super-users and regular forum members, as well as people who have been invited to join an online forum but not done so.

Interview topic guides will include an opportunity for participants to share what they understand the role of online forums to be, their impacts, and how they “work”. With our moderators, host and commissioners we will also explore their understanding of the role of moderators, how they make decisions regarding moderating behaviours, and how they are trained and supported in this role. Interview topic guides will evolve to specifically test our hypotheses. Interviews will be face-to-face or via secure video conferencing (Microsoft Teams), or telephone, recorded and transcribed. Analysis will first be done within case, and then integrated to compare between cases. Data will be managed in NVIVO. Consistent with the realist approach, analysis will be retroductive i.e., seek to identify the hidden causal forces underlying people’s descriptions of their experiences in the forums. These will be initially coded into our hypothesised CMO configurations from Workstream 1, but the analysis will be developed through regular discussion with the research team and PPI/Codesign group.

Analysis of forum posts

The collection of posts from our online forums will result in large linguistic datasets, potentially amounting to millions of words in some cases. To analyse these, we will use Corpus-based Discourse Analysis and Natural Language Processing (50). We will first describe the size, shape, content, emotional tone and linguistic features of each of our forum cases, also considering how each of these features change over time. In the second stage, we will adapt methods to test our specific CMO configurations. Specific techniques we can draw on to do this are summarised in Table 1. Analyses will be carried out using the latest versions of cutting-edge corpus tools developed at Lancaster University (#LancsBox, the Lancaster Stats Tools Online, lexiDB and Wmatrix), and, where appropriate, the corpus analysis tools available in AntLab, and Sketch Engine and state-of-the-art NLP libraries and repositories such as Spacy and Hugging Face.

Table 1- Overview of computational linguistic techniques to analyse forum posts

Stage 1 technique	Function
Frequency and dispersion analyses	To obtain the frequencies and distribution of selected words, phrases or semantic domains in a corpus and its sub-sections.

For peer review only

Keyness analyses	To identify and study the words, phrases and concepts characteristic of a particular (sub-)corpus, e.g.: an online community vs. a general corpus of English; moderated vs. unmoderated communities; first posts vs. last posts; patients' posts vs. moderators' posts; etc.
Change over time analyses	To track changes in co-occurring words of critical interest over time to capture continuities and discontinuities in meanings and associations
Summarisation or topic modelling	To generate short summaries of forums or parts of forums and locate the most important posts.
Social network analysis	To extract the structure and shape of the network of people in the community, to see who is talking to whom, to classify user's participation style (observers, users, super-users).
User profiling	To estimate the relative usage and representativeness of people in each community by age and gender, country location and other demographic variables
**Stage 2 technique**	**Function**
Concordance analyses	To obtain all occurrences of selected words, phrases or instances of different semantic domains, and to understand how and why they are used, e.g.: words such as 'feel', 'daughter', and 'medication' or semantic domains such as 'Sad', 'Personal relationships' and 'Medicines and medical treatment'.
Collocation analyses	To study patterns of co-occurrence of words or phrases, which are well known to contribute to their meanings and associations, e.g. what words tend to co-occur with 'you', 'not' or 'worry' in different (sub-)corpora.
Sentiment analysis	To study change in emotion over time and across forums or threads, showing positive and negative emotions, and we will combine this with an analysis of risk indicator words and taboo words, and terms of emotional support or bullying.

Qualitative analyses will be employed to carry out in-depth studies of specific posts, interactions or threads selected to test our specific theories. This might include threads with particularly high or low frequencies of negatively valanced emotion words; posts or threads that were found to be prototypical of a whole forum in linguistic terms; posts that received large numbers of replies; posts by individuals representing different levels of engagement or roles with the online forum, or showing evidence of significant change in emotional state. The qualitative analyses will investigate how users and moderators express and interactively manage identities, experiences, knowledge, emotions and relationships, and with what local impacts in terms of patterns in responses and interactional behaviour. These analyses will be directed at testing our CMOs by exploring users' understandings of mechanisms underlying the impact that participation in the online forum has on themselves and others.

Triangulation of data across methods and between cases

All methods have limitations, and in combining our survey, interviews, and analysis of forum posts, we can ensure that: we have a full range of perspectives (including those who choose not to take part in all aspects); we can purposively sample for interview on the basis of self-reported and real time evidence of specific impacts, or membership to specific subgroups; we can move between broad contextual data, such as the size and structure of the forum, to detailed analysis of individual conversations: we can test specific programme theories in multiple ways to test reliability and validity of findings. Regular analysis meetings, including our PPI/Codesign group, will ensure that we

For peer review only

make the most of the opportunity for iterative and integrated analysis across our cases and between our researchers using different methods.

Workstream 3 (Months 2-34) Impact

Our theories will be used to underpin the co-design of best-practice tools and support for people involved in commissioning, hosting, moderating or using online mental health forums (**RQ3**). All tools will be informed by the programme theory developed in Workstreams 1 & 2. The exact nature of the tools will depend on the outcomes of the co-design process, but our pre-application work with our PPI, hosts and impact groups has suggested the candidate tools described in Table 2.

We will design all outputs and our dissemination strategy to be accessible across age, gender, ethnicity, disability and the digital divide. We will write plain English summaries, translated into other languages where possible, and disseminate via non-digital platforms (in person, radio, & print media) as well as digitally (websites, social media etc).

Table 2. Candidate tools and implementation

Tool	Aim
For people with mental health difficulties, referrers, and commissioners	
Knowledge exchange video/animation tools	Widening access and promoting greater uptake through better understanding of the role of online mental health forums and how they work. These tools will describe how online forums work, possible benefits, for who, and how to identity safe and supportive forums.
For forum moderators	
Community of Practice (CoP)	Building capacity through facilitating mutual engagement, joint enterprise, and shared practice. Such support is crucial to continuing professional development, and to prevent compassion fatigue and burnout. Openly available to all the moderators involved in our study and providing a safe online space for the forum to evolve from the outset of the study.
E-learning curricula	To train and support moderators in reflexive practice including: understanding the moderator role; ethics of moderation; encouraging activity; understanding mental health; spotting moments of change (introductory linguistic analysis); managing challenging situations; widening access and welcoming diversity; identifying and managing risks; looking after yourself; role of supervision & peer support; signposting; continuing professional development

For peer review only

	(CPD). The content will draw on challenges described by moderators during our interviews, and practical case examples of ways to manage these.
For forum providers/hosts, commissioners and policy makers	
Best practice design principles for online mental health forums	To widen access, maximise positive impacts, and minimise harms. This guidance will provide 1) High level design principles to guide forum design and moderation. 2) Practical guidance for implementing design features to improve usefulness and safety of online forums 3) Examples of best practice and case studies on forum design and moderation practices.
Resource requirements for implementation of best practice design principles based on the disaggregation of the programme theories into resources and reasoning	This will be based on the disaggregation of the programme theories into resources and reasoning and will provide information on the resource configuration necessary to provide safe and effective online community.
Standardised forum characterisation and evaluation framework	To support standardised evaluation and comparison of forums during commissioning
Methodological guidance on how to evaluate online mental health forums	To provide guidance for evaluation of efficacy and effectiveness
For further theory and practice development	
Programme theory	To generate a theory that is generalisable to online mental health forums and with relevance to understanding online peer support interventions across healthcare.

Knowledge Mobilisation

As the host site for this study, Berkshire Healthcare NHS Foundation Trust will take a lead role in ensuring the best practice tools outputs will be easily discovered by our relevant audiences, openly accessible, free to use, and updated and maintained beyond the life of the project. Ongoing feedback and revisions will be built into the design to ensure these tools evolve as needed. We will also work with the Health Education England learning hub (https://learninghub.nhs.uk), NHS England, ORCHA (https://orchahealth.com) and Oxford AHSN to explore how these tools can be best designed and promoted across moderators working across NHS, charity and commercial organisations.

Discussion

Online mental health forums have the potential to greatly increase access to peer support, but the impacts of using forums, and the underlying mechanisms, need to be better understood. This protocol outlines a methodologically novel triangulation of survey, interview and corpus linguistic methods, to generate and test realist programme theories about how online forums "work" for forum users and moderators, and to use these theories to develop best policy and practice tools to improve peer online forums. This study will be an inherently iterative process, and therefore the aim

For peer review only

of the protocol is not to prospectively define all our decisions at the outset, but rather to explore some of the key issues that have arisen and to share our learning from the challenges we have faced in designing the protocol.

The first challenge was to develop an ethical framework for the use of online forums posts as research data. We adopted a user centred approach, considering a user's likely expectations and concerns; consulted health forum hosts, moderators and users; hosted an online focus group (N = 21) exploring the study's benefits and risks and how best to mitigate these; examined key guidance on using online data including a) the British Psychological Society (42) and b) Association of internet researchers (51); collaborated with legal experts at Berkshire Healthcare NHS Foundation Trust and the information officer at Lancaster University, and discussed our strategy widely to learn how other researchers in the field have managed these ethical issues. The outcome of this process is described in our Data Processing Plan shown in Table 3 below and published on our study website (www.lancaster.ac.uk/ipof/ethics-framework). The process raised several interesting ethical issues which will be described in detail in a separate paper.

The second challenge was to build a team of collaborators, each expert in their own discipline, but not all familiar with the realist approach. Statistical analysis of surveys, reflexive thematic analysis of interview transcripts, corpus linguistic analysis of forum posts, and realist synthesis of published literature, all originate from different philosophical and theoretical positions, and vary in the extent to which they use different styles of reasoning (inductive, deductive, abductive, retroductive etc.). We have divided the project into workstreams which focus initially on the set up and descriptive analysis of the survey, interview and linguistic data. However, we have also built a collaborative workstream throughout the project to focus on developing a shared understanding of the realist approach, to codevelop the programme theories, and codesign novel integrated methods to test these.

The final challenge has been establishing our PPI/Codesign group. We wanted to ensure moderators and forum members for all our participating forums had a significant ongoing role throughout the project. They have the expertise to ensure the study is designed and conducted in a way that is engaging and acceptable to participants (PPI work), and the outputs are designed to be useful and effective (codesign participation). We have planned for this group to meet monthly throughout the study. However, despite increasingly detailed guidance from the NIHR about payment for involvement (52), this has been a challenge. Due to the extent and longevity of involvement, PPI members are required to be employees of the University, to share evidence of their right to work in the UK and navigate a bureaucratic contract-based payment process. These payment methods were not designed for the inherently flexible and iterative nature of PPI co-design work described above. Fear of losing access to benefits due to being deemed able to work as a result of getting involved in this kind of project has been another barrier to engagement. This can exclude those from more marginalised groups, whose voices need to be heard in this kind of research.

Ethics and Dissemination

Ethical approval was granted by Solihull Research Ethics Committee (**IRAS 314029**) on the 20th of June 2022. Table 3 describes each step in the process from data collection to dissemination for each of the three different types of data being collected.

Table 3 – Data Processing Plan for iPOF.

	**Forum Data**	**Survey Data**	**Interview Data**

**Collection**	Posts from publicly available forums with no expectation of privacy, where the forum host consents, will be accessed directly. Prior to downloading the data, the study will be described and users invited to withdraw their data if they wish.<br>Posts from forums requiring a login, with an expectation of privacy, will only be used from individuals who have freely consented (i.e. they had the option to not consent and still use the service) to their data being used for research. Prior to downloading the data, the study will be described, and users invited to have their data removed if they wish.<br>Posts from forums in which consent for data to be used for research is required before joining the forum, will be used, but only where it is possible to ensure all users have been made aware of the option to opt out.<br>Posts from forums linked to health or social care records, and no consent has been given for research at sign up, will only be used with additional individual informed consent.<br>Finally, posts from forums that are closed, publicly available, and where there are no links between posts and any personal identifiable data will be used without additional consent.	Using REDCap with individual informed consent.	Using Microsoft Teams and an encrypted audio recorder, with individual informed consent
**De-Identification**	When forums are anonymous and publicly available, we will replace all usernames with a PIN and automatically remove any names of places or people that could be identifiable.<br>For all other forums, posts will be deidentified by the host organisation before being shared with us.	Identifiable data, such as on consent forms, will be stored separately to the survey results.	Identifying information will be removed from transcripts. Identifiable data, such as on consent forms, will be stored separately.
**Transfer**	Data transfer from online communities to Lancaster University will be secure and encrypted (e.g. secure FTP and HTTPS).	Data will be collected via a link directly into Lancaster University system.	Audio files will be uploaded to Lancaster's servers and deleted from the recorder. Transcription will be done by a University approved and contracted transcriber.
**Storage**	Using Lancaster University's approved IT systems.	Data will be stored on Lancaster University's secure servers.	Recordings and transcripts will be stored on Lancaster University's secure servers.
**Analysis**	Analysis (Natural Language Processing) will be conducted by methodological expert members of the research team. (See Table 1)	Analyses will include detailed description of the sample and the use of generalized mixed models and structural	Analysis will be retroductive and will contribute to hypothesised CMO

For peer review only

		equation models using Mplus (v8.6).	configurations. Analysis will be managed in NVIVO.
**Deletion**	Participants will have the right to withdraw and request that their data be deleted, up to the analysis starting.	Participants have 1 week to request their data to be removed.	Participants will have the right to withdraw and request that their data be deleted, up to the analysis starting.
**Publication**	To preserve anonymity, paraphrased forum quotes will be published.	Minimum cell sizes will be adopted for published results.	If consent is given, direct quotes will be published. Any potentially identifying information will be removed.
**Archiving & Access**	All papers will be published open access. Given the nature of the data we will not share the forum datasets openly.	Deidentified survey data will be openly shared on PURE.	Interview data will be restricted access and available by request to legitimate research parties if the purpose is consistent with the consent given for this research.

We will define our range of audiences, identify what each needs to know, and prepare targeted outputs appropriately created and written for this audience including Plain English summaries. Findings will be reported in accordance with RAMESES (53) guidelines, published as open access and shared widely, along with codesigned tools. Study outputs will be promoted through a national launch of the co-designed tools, as part of a conference, webinar, and social media strategy. Our protocols and academic findings will be published in open access peer reviewed journals and presented at academic and peer forum conferences. All study participants and relevant health, social, educational and academic organisations will be kept updated via a study website (https://www.lancaster.ac.uk/ipof), social media (twitter), and blogs from across the wider team. All policy and practice guidance will be hosted, updated, and made widely available via Berkshire Healthcare NHS Foundation Trust.

Contributorship statement

All authors contributed to drafting the manuscript and approved the final version. The study was conceived by FL, developed for funding by FL, JRM, ES, NS, PR, SJ, CL, RM, SW, MR, and further adapted in the setup phase by input from all authors.

Theory development and testing is led by PM, HR, and MC, with guidance in realist methodology from JRM. Qualitative analysis of interviews and corpus linguistics is led by ES; computational analysis by PR and MC; survey design and analysis by NS; codesign by SJ, and KM; PPI by CL. The programme is managed by TR, with research administration support from RJ; ZG; KP, and ED. Clinical input is provided by SJ; FL; SW and JH.

Competing Interests statement

The authors declare no competing interests.

Funding Statement

This study is funded by the NIHR Health and Social Care Delivery Research (HS&DR) (NIHR134035). The views expressed are those of the author(s) and not necessarily those of the NIHR or the Department of Health and Social Care. The study is hosted by Berkshire Healthcare NHS Foundation Trust. The Sponsor is Lancaster University. Additional support comes from the NIHR Clinical Research Network, NIHR North-West Coast Applied Research Collaboration (ARC), and Oxford Allied Health Sciences Network (AHSN).

For peer review only

Data sharing statement

No data are available.

References

1. McManus S, Bebbington PE, Jenkins R, Brugha T. Mental Health and Wellbeing in England: the Adult Psychiatric Morbidity Survey 2014: NHS digital; 2016.
2. NHS Confederation. Digital inclusion in mental health - a guide to help increase choice and improve access to digital mental health services. 2020.
3. Ofcom. Children and parents: Media use and attitudes report 2019. Ofcom London, UK; 2020.
4. Saha K, Sharma A, editors. Causal factors of effective psychosocial outcomes in online mental health communities. Proceedings of the International AAAI Conference on Web and Social Media; 2020.
5. Hanley T, Prescott J, Gomez KU. A systematic review exploring how young people use online forums for support around mental health issues. Journal of Mental Health. 2019;28(5):566-76.
6. Naslund J, Aschbrenner K, Marsch L, Bartels S. The future of mental health care: peer-to-peer support and social media, Epidemiology and Psychiatric Sciences, 25, 113-122. 2016.
7. Coulson NS, Bullock E, Rodham K. Exploring the therapeutic affordances of self-harm online support communities: An online survey of members. JMIR mental health. 2017;4(4):e44.
8. Brady E, Segar J, Sanders C. "I always vet things": navigating privacy and the presentation of self on health discussion boards among individuals with long-term conditions. Journal of medical Internet research. 2016;18(10):e274.
9. Griffiths KM, Reynolds J, Vassallo S. An online, moderated peer-to-peer support bulletin board for depression: user-perceived advantages and disadvantages. JMIR Mental Health. 2015;2(2):e14.
10. Powell J, McCarthy N, Eysenbach G. Cross-sectional survey of users of Internet depression communities. BMC psychiatry. 2003;3(1):19.
11. Prescott J, Rathbone AL, Hanley T. Online mental health communities, self-efficacy and transition to further support. Mental Health Review Journal. 2020.
12. Bartlett YK, Coulson NS. An investigation into the empowerment effects of using online support groups and how this affects health professional/patient communication. Patient education and counseling. 2011;83(1):113-9.
13. Smith-Merry J, Goggin G, Campbell A, McKenzie K, Ridout B, Baylosis C. Social connection and online engagement: insights from interviews with users of a mental health online forum. JMIR mental health. 2019;6(3):e11084.
14. Griffiths KM, Mackinnon AJ, Crisp DA, Christensen H, Bennett K, Farrer L. The effectiveness of an online support group for members of the community with depression: a randomised controlled trial. PloS one. 2012;7(12):e53244.
15. Marchant A, Hawton K, Stewart A, Montgomery P, Singaravelu V, Lloyd K, et al. A systematic review of the relationship between internet use, self-harm and suicidal behaviour in young people: The good, the bad and the unknown. PloS one. 2017;12(8):e0181722.
16. Dunlop SM, More E, Romer D. Where do youth learn about suicides on the Internet, and what influence does this have on suicidal ideation? Journal of child psychology and psychiatry. 2011;52(10):1073-80.
17. Moore D, Ayers S. Virtual voices: social support and stigma in postnatal mental illness Internet forums. Psychology, health & medicine. 2017;22(5):546-51.
18. Rodgers RF, Lowy AS, Halperin DM, Franko DL. A meta-analysis examining the influence of pro-eating disorder websites on body image and eating pathology. European Eating Disorders Review. 2016;24(1):3-8.

For peer review only

19. Bailey E, Robinson J, Alvarez-Jimenez M, Nedeljkovic M, Valentine L, Bendall S, et al. Moderated online social therapy for young people with active suicidal ideation: qualitative study. Journal of medical internet research. 2021;23(4):e24260.
20. Easton K, Diggle J, Ruethi-Davis M, Holmes M, Byron-Parker D, Nuttall J, et al. Qualitative Exploration of the Potential for Adverse Events When Using an Online Peer Support Network for Mental Health: Cross-Sectional Survey. JMIR mental health. 2017;4(4):e49.
21. Ziebland S, Wyke S. Health and illness in a connected world: how might sharing experiences on the internet affect people's health? The Milbank Quarterly. 2012;90(2):219-49.
22. Malik S, Coulson NS. 'They all supported me but I felt like I suddenly didn't belong anymore': an exploration of perceived disadvantages to online support seeking. Journal of Psychosomatic Obstetrics & Gynecology. 2010;31(3):140-9.
23. Lobban F, Appelbe D, Appleton V, Billsborough J, Fisher NR, Foster S, et al. IMPlementation of An online Relatives' Toolkit for psychosis or bipolar (IMPART study): iterative multiple case study to identify key factors impacting on staff uptake and use. BMC health services research. 2020;20(1):1-13.
24. Lobban F, Appelbe D, Appleton V, Aref-Adib G, Barraclough J, Billsborough J, et al. An online supported self-management toolkit for relatives of people with psychosis or bipolar experiences: the IMPART multiple case study. Health Services and Delivery Research. 2020;8(37).
25. Krasodomski-Jones A. Everything in moderation: platforms, communities and users in a healthy online environment.
26. Sibilla F, Mancini T. I am (not) my avatar: A review of the user-avatar relationships in Massively Multiplayer Online Worlds. Cyberpsychology: Journal of Psychosocial Research on Cyberspace. 2018;12(3).
27. Suler J. The online disinhibition effect. Cyberpsychology & behavior. 2004;7(3):321-6.
28. SMITH J, KRASODOMSKI-JONES A, OLANIPEKUN M, JUDSON E. A picture of health march 2021: Measuring the comparative health of online spaces. 2021.
29. Grimmelmann J. The virtues of moderation. Yale JL & Tech. 2015;17:42.
30. Smith J, Jones E, Judson E. What's in a name? A forward view of anonymity online.
31. Yin RK. Case study research and applications: Design and methods: Sage publications; 2017.
32. Staniszewska S, Brett J, Simera I, Seers K, Mockford C, Goodlad S, et al. GRIPP2 reporting checklists: tools to improve reporting of patient and public involvement in research. bmj. 2017;358.
33. Pawson R, Greenhalgh T, Harvey G, Walshe K. Realist synthesis: an introduction. Manchester: ESRC Research Methods Programme, University of Manchester. 2004.
34. Rycroft-Malone J, McCormack B, Hutchinson AM, DeCorby K, Bucknall TK, Kent B, et al. Realist synthesis: illustrating the method for implementation research. Implementation Science. 2012;7(1):33.
35. Jagosh J. Realist synthesis for public health: building an ontologically deep understanding of how programs work, for whom, and in which contexts. Annual review of public health. 2019.
36. Hunter R, Gorely T, Beattie M, Harris K. Realist review. International Review of Sport and Exercise Psychology. 2022;15(1):242-65.
37. Wadden D, August T, Li Q, Althoff T. The Effect of Moderation on Online Mental Health Conversations. arXiv preprint arXiv:200509225. 2020.
38. Bonell C, Jamal F, Melendez-Torres G, Cummins S. 'Dark logic': theorising the harmful consequences of public health interventions. J Epidemiol Community Health. 2015;69(1):95-8.
39. Kaylor-Hughes CJ, Rawsthorne M, Coulson NS, Simpson S, Simons L, Guo B, et al. Direct to public peer support and e-therapy program versus information to aid self-management of depression and anxiety: protocol for a randomized controlled trial. JMIR research protocols. 2017;6(12):e231.
40. Powell J, Hamborg T, Stallard N, Burls A, McSorley J, Bennett K, et al. Effectiveness of a web-based cognitive-behavioral tool to improve mental well-being in the general population: randomized controlled trial. Journal of medical Internet research. 2013;15(1):e2.

For peer review only

41. Patridge E, Bardyn T. Research Electronic Data Captrue (REDCap). Journal of the Medical Library Association. 2018;106(1):142-4.
42. Hewson C, Buchanan T, editors. Ethics guidelines for internet-mediated research2013: The British Psychological Society.
43. Spitzer RL, Kroenke K, Williams JB, Löwe B. A brief measure for assessing generalized anxiety disorder: the GAD-7. Archives of internal medicine. 2006;166(10):1092-7.
44. Kroenke K, Spitzer RL, Williams JB. The PHQ-9: validity of a brief depression severity measure. Journal of general internal medicine. 2001;16(9):606-13.
45. Maxwell SE, Cole DA. Bias in cross-sectional analyses of longitudinal mediation. Psychological methods. 2007;12(1):23.
46. Rabe-Hesketh S, Skrondal A, Pickles A. Generalized multilevel structural equation modeling. Psychometrika. 2004;69(2):167-90.
47. Pan H, Liu S, Miao D, Yuan Y. Sample size determination for mediation analysis of longitudinal data. BMC Medical Research Methodology. 2018;18(1):1-11.
48. Pawson R. Theorizing the interview. British Journal of Sociology. 1996:295-314.
49. Manzano A. The craft of interviewing in realist evaluation. Evaluation. 2016;22(3):342-60.
50. Baker P, Gabrielatos C, Khosravinik M, Krzyżanowski M, McEnery T, Wodak R. A useful methodological synergy? Combining critical discourse analysis and corpus linguistics to examine discourses of refugees and asylum seekers in the UK press. Discourse & society. 2008;19(3):273-306.
51. Research AoI. Ethics: Association of Internet Research; [Available from: https://aoir.org/ethics/.
52. NIHR. Payment guidance for researchers and professionals. NIHR. 2021.
53. The RAMESES Projects 2022 [Available from: https://www.ramesesproject.org/.

For peer review only

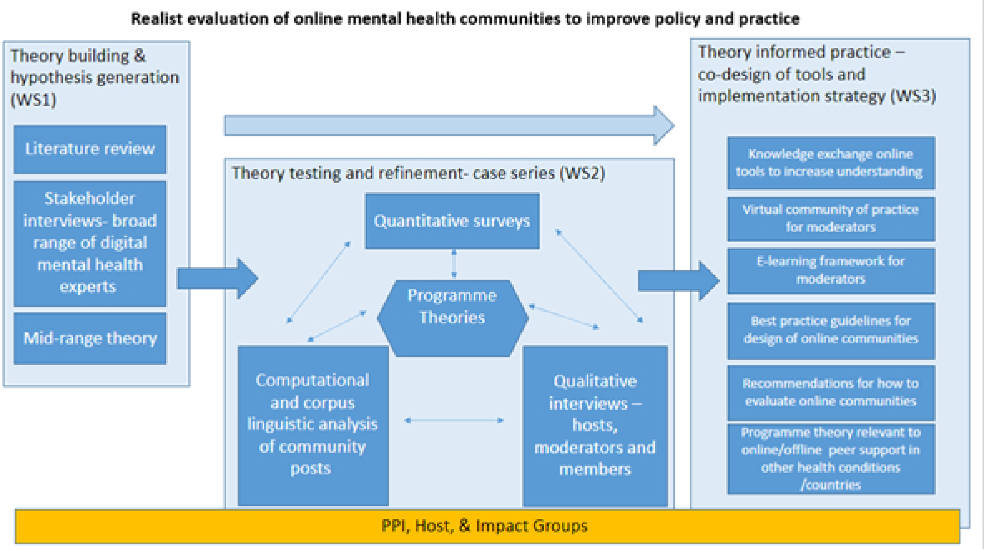

402x221mm (38 x 38 DPI)

Explore journals Get published About BMC Login
Search
Menu

BMC Medicine

Home About Articles Submission Guidelines Submit manuscript

Table 1 List of items to be included when reporting a realist synthesis

From: RAMESES publication standards: realist syntheses

TITLE		
1		In the title, identify the document as a realist synthesis or review
ABSTRACT		
2		While acknowledging publication requirements and house style, abstracts should ideally contain brief details of: the study's background, review question or objectives; search strategy; methods of selection, appraisal, analysis and synthesis of sources; main results; and implications for practice.
INTRODUCTION		
3	Rationale for review	Explain why the review is needed and what it is likely to contribute to existing understanding of the topic area.
4	Objectives and focus of review	State the objective(s) of the review and/or the review question(s). Define and provide a rationale for the focus of the review.
METHODS		
5	Changes in the review process	Any changes made to the review process that was initially planned should be briefly described and justified.
6	Rationale for using realist synthesis	Explain why realist synthesis was considered the most appropriate method to use.
7	Scoping the literature	Describe and justify the initial process of exploratory scoping of the literature.
8	Searching processes	While considering specific requirements of the journal or other publication outlet, state and provide a rationale for how the iterative searching was done. Provide details on all the sources accessed for information in the review. Where searching in electronic databases has taken place, the details should include, for example, name of database, search terms, dates of coverage and date last searched. If individuals familiar with the relevant literature and/or topic area were contacted, indicate how they were identified and selected.

For peer review only - http://bmjopen.bmj.com/site/about/guidelines.xhtml

TITLE		
9	Selection and appraisal of documents	Explain how judgements were made about including and excluding data from documents, and justify these.
10	Data extraction	Describe and explain which data or information were extracted from the included documents and justify this selection.
11	Analysis and synthesis processes	Describe the analysis and synthesis processes in detail. This section should include information on the constructs analyzed and describe the analytic process.
RESULTS		
12	Document flow diagram	Provide details on the number of documents assessed for eligibility and included in the review with reasons for exclusion at each stage as well as an indication of their source of origin (for example, from searching databases, reference lists and so on). You may consider using the example templates (which are likely to need modification to suit the data) that are provided.
13	Document characteristics	Provide information on the characteristics of the documents included in the review.
14	Main findings	Present the key findings with a specific focus on theory building and testing.
DISCUSSION		
15	Summary of findings	Summarize the main findings, taking into account the review's objective(s), research question(s), focus and intended audience(s).
16	Strengths, limitations and future research directions	Discuss both the strengths of the review and its limitations. These should include (but need not be restricted to) (a) consideration of all the steps in the review process and (b) comment on the overall strength of evidence supporting the explanatory insights which emerged.<br><br>The limitations identified may point to areas where further work is needed.
17	Comparison with existing literature	Where applicable, compare and contrast the review's findings with the existing literature (for example, other reviews) on the same topic.
18	Conclusion and recommendations	List the main implications of the findings and place these in the context of other relevant literature. If appropriate, offer recommendations for policy and practice.
19	Funding	Provide details of funding source (if any) for the review, the role played by the funder (if any) and any conflicts of interests of the reviewers.

For peer review only

Back to article page ›

BMC Medicine

ISSN: 1741-7015

Contact us

Submission enquiries: bmcmedicineeditorial@biomedcentral.com
