# Supplementary material for: Improving Peer Online Forums (iPOF): protocol for a realist evaluation of peer online mental health forums to inform practice and policy
Source: BMJ Open. 2023 Jul 30;13(7):e075142. doi: 10.1136/bmjopen-2023-075142 (PMC10387651; doi:10.1136/bmjopen-2023-075142)
Supplement: Reviewer comments [file bmjopen-2023-075142.reviewer_comments.pdf]

PEER REVIEW HISTORY

BMJ Open publishes all reviews undertaken for accepted manuscripts. Reviewers are asked to complete a checklist review form (**http://bmjopen.bmj.com/site/about/resources/checklist.pdf**) and are provided with free text boxes to elaborate on their assessment. These free text comments are reproduced below.

ARTICLE DETAILS

**TITLE (PROVISIONAL)**	Improving Peer Online Forums (iPOF): Protocol for a Realist Evaluation of Peer Online Mental Health Forums to Inform Practice and Policy
**AUTHORS**	Lobban, Fiona; Coole, Matthew; Donaldson, Emma; Glossop, Zoe; Haines, Jade; Johnston, Rose; Jones, Steven; Lodge, Christopher; Machin, Karen; Marshall, Paul; Meacock, Rachel; Penhaligon, Kate; Rakić, Tamara; Rawsthorne, Mat; Rayson, Paul; Robinson, Heather; Rycroft-Malone, Jo; Semino, Elena; Shryane, Nick; Wise, Sara

This article was not externally reviewed at BMJ Open. Protocols that have been independently assessed prior to submission to BMJ Open are usually fast-tracked to publication on the grounds that further substantial changes will not be possible. This independent assessment will usually be external, independent review for both a substantial grant award from a non-commercial or government funder and ethics approval.
